# Environmental fatty acids enable emergence of infectious *Staphylococcus aureus* resistant to FASII-targeted antimicrobials

**DOI:** 10.1038/ncomms12944

**Published:** 2016-10-05

**Authors:** Claire Morvan, David Halpern, Gérald Kénanian, Constantin Hays, Jamila Anba-Mondoloni, Sophie Brinster, Sean Kennedy, Patrick Trieu-Cuot, Claire Poyart, Gilles Lamberet, Karine Gloux, Alexandra Gruss

**Affiliations:** 1 Micalis Institute, INRA, AgroParisTech, Université Paris-Saclay, Jouy-en-Josas 78350, France; 2 ‘Barriers and Pathogens' Team, INSERM U 1016, Institut Cochin, Paris F-75014, France; 3 CNRS UMR 8104, Paris F-75014, France; 4 Université Paris Descartes, Sorbonne Paris Cité, Paris F-75014, France; 5 Hôpitaux Universitaires Paris Centre Cochin-Hôtel Dieu-Broca, Assistance Publique Hôpitaux de Paris, Paris F-75014, France; 6 Metagenopolis-Micalis UMR 1319, Jouy en Josas 78352, France; 7 Biology of Gram-positive Pathogens Unit. Institut Pasteur 25-28 rue du docteur Roux, Paris 75015, France; 8 CNRS ERL3526, Paris 75015, France

## Abstract

The bacterial pathway for fatty acid biosynthesis, FASII, is a target for development of new anti-staphylococcal drugs. This strategy is based on previous reports indicating that self-synthesized fatty acids appear to be indispensable for *Staphylococcus aureus* growth and virulence, although other bacteria can use exogenous fatty acids to compensate FASII inhibition. Here we report that staphylococci can become resistant to the FASII-targeted inhibitor triclosan *via* high frequency mutations in *fabD*, one of the FASII genes. The *fabD* mutants can be conditional for FASII and not require exogenous fatty acids for normal growth, and can use diverse fatty acid combinations (including host fatty acids) when FASII is blocked. These mutants show cross-resistance to inhibitors of other FASII enzymes and are infectious in mice. Clinical isolates bearing *fabD* polymorphisms also bypass FASII inhibition. We propose that fatty acid-rich environments within the host, in the presence of FASII inhibitors, might favour the emergence of staphylococcal strains displaying resistance to multiple FASII inhibitors.

## Abstract (web summary)

The bacterial pathway for fatty acid biosynthesis, FASII, is a target for development of new anti-staphylococcal drugs. Here, Morvan *et al*. show that exogenous fatty acids can favour the emergence of staphylococcal strains displaying resistance to multiple FASII inhibitors.

## Graphical abstract

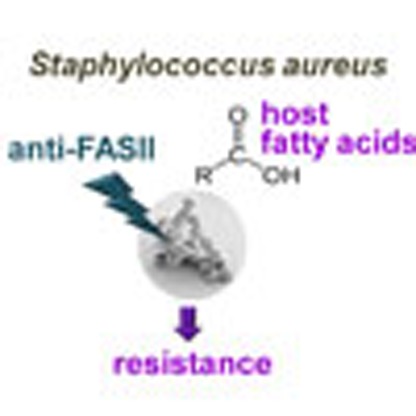

S*taphylococcus* aureus is a remarkably versatile opportunist pathogen that has confounded the medical community by its ability to escape antibiotic inhibition. Multidrug-resistant *S. aureus* are a main cause of hospital- and community-acquired infections, and treatment constitutes a major medical and economic challenge^1^,^2^. The fatty acid synthesis pathway (FASII) has been a leading candidate as a novel essential target for antimicrobial discovery. Numerous FASII inhibitors are in the pipeline or in clinical trials, most of which target FabI, the enoyl-acyl carrier protein (ACP) reductase (for example, AFN-1252 (now Debio-1452 and pro-drug Debio-1450), CG400549, CG400462 and MUT056399), or FabF, the β-ketoacyl-ACP synthase (for example, platencin, platensimycin and fasamycins A and B)^3^,^4^,^5^,^6^,^7^,^8^,^9^,^10^,^11^.

Triclosan, a chemically synthesized biocide first introduced in 1965, is the best known FabI inhibitor developed thus far, and has had widespread use in hygiene, health care, and household products at concentrations of up to 1% (refs 12, 13). In consequence, triclosan detection in human fluids is common, and has generated information on the limits of use of this anti-FASII, including safety issues^14^,^15^,^16^,^17^,^18^,^19^. Observed side effects of triclosan on the host may be caused by its general membrane-disrupting activity^12^,^17^,^19^. Additionally, triclosan-resistant staphylococci, conferred by mutations that maintain FASII activity, or obtained by horizontal transfer of the *Staphylococcus haemolyticus fabI* gene (encoding a naturally occurring resistant FabI variant), are estimated to comprise 5–15% of *S. aureus* clinical isolates^14^,^16^,^20^. Continued anti-FASII development is based on development of antimicrobials with greater FabI affinity and specificity that would curb its potentially dangerous side effects^4^,^9^,^21^,^22^,^23^,^24^.

However, fatty acids are abundant in the host and can be used by several Gram-positive pathogens to overcome a FASII deficiency^25^,^26^,^27^,^28^, which questions FASII targeting as a general strategy for antibacterial drug discovery. For example, exogenous fatty acids can fully compensate a FASII deficiency in *Streptococcus agalactiae*, and a FASII auxotroph was fully virulent in a murine septicaemic infection model^25^.

Nevertheless, the ability of *S. aureus* to override FASII inhibition in the presence of fatty acids has remained in question^4^,^21^,^26^,^29^,^30^. *S. aureus* FASII expression is not blocked by exogenous fatty acids, whereas it is inhibited in streptococci^21^. Rather than synthesizing unsaturated fatty acids to fluidify their membranes (as do the streptococci), *S. aureus* synthesizes branched chain fatty acids, of which the main one, anteiso C15:0 (*ai*15:0), is apparently required for phospholipid synthesis^27^,^31^,^32^. Moreover, it has been proposed that FASII inhibition in *S. aureus* leads to accumulation of acyl-ACP intermediates, thereby limiting ACP availability for exogenous fatty acid utilization^9^,^27^,^33^,^34^ (see Supplementary Figs 1 and 2 for pathway and genetic contexts). In addition, while fatty acid auxotrophs (defective in *acc* genes encoding acetyl-CoA carboxylase) are viable, optimal growth requires *ai*15:0, and these *acc* mutants are avirulent in a mouse septicaemic infection model^24^,^27^,^32^.

The fundamental and medical importance of FASII-targeted drug development incited us to characterize *S. aureus* responses to anti-FASII drugs. Here we show that fatty acids, which are ubiquitous host constituents, can facilitate the emergence of *S. aureus* resistance to anti-FASII drugs.

## Results

### Fatty acids facilitate the emergence of triclosan resistance

Some Gram-positive bacteria can incorporate exogenous fatty acids, which enable resistance to anti-FASII drugs^25^,^26^. In the case of *S. aureus*, we considered the possibility that arising mutations could facilitate fatty acid incorporation and lead to FASII bypass, as hypothesized in an earlier study^27^. To reveal such fatty-acid-based triclosan resistant (FA-T^R^) mutants, we examined the response of the *S. aureus* Newman strain to the FabI inhibitor triclosan on fatty-acid-containing BHI solid medium. A fatty acid cocktail (referred hereafter as ‘FA') was used comprising C14:0, C16:0, and C18:1, which are present in the human host^35^,^36^. Overnight *S. aureus* BHI cultures were plated on medium containing FA and 0.25 μg ml^−1^ triclosan; as controls, they were also plated on medium without FA and/or triclosan. Triclosan-resistant colonies arose at about 100-fold higher frequencies (∼10^−6^) in the presence of FA. The FA-T^R^ colonies were screened for growth on BHI, BHI-triclosan (BHI-T), BHI-FA and BHI-FA-triclosan (BHI-FA-T) plates (Supplementary Fig. 3). A minority of clones grew on all four media (FA prototroph triclosan resistant mutants, T^R^ mutants). The remaining clones grew either on BHI, BHI-FA and BHI-FA-T (FA prototrophs), or on FA-containing media but very poorly or not at all on BHI. The latter were thus considered as fatty-acid-dependent for normal growth and were designated DepT^R^ mutants. Fatty acids thus appear to facilitate the emergence of triclosan resistance in *S. aureus*.

### Three classes of *S. aureus* FA-T^R^ mutants

**Figure 1:**
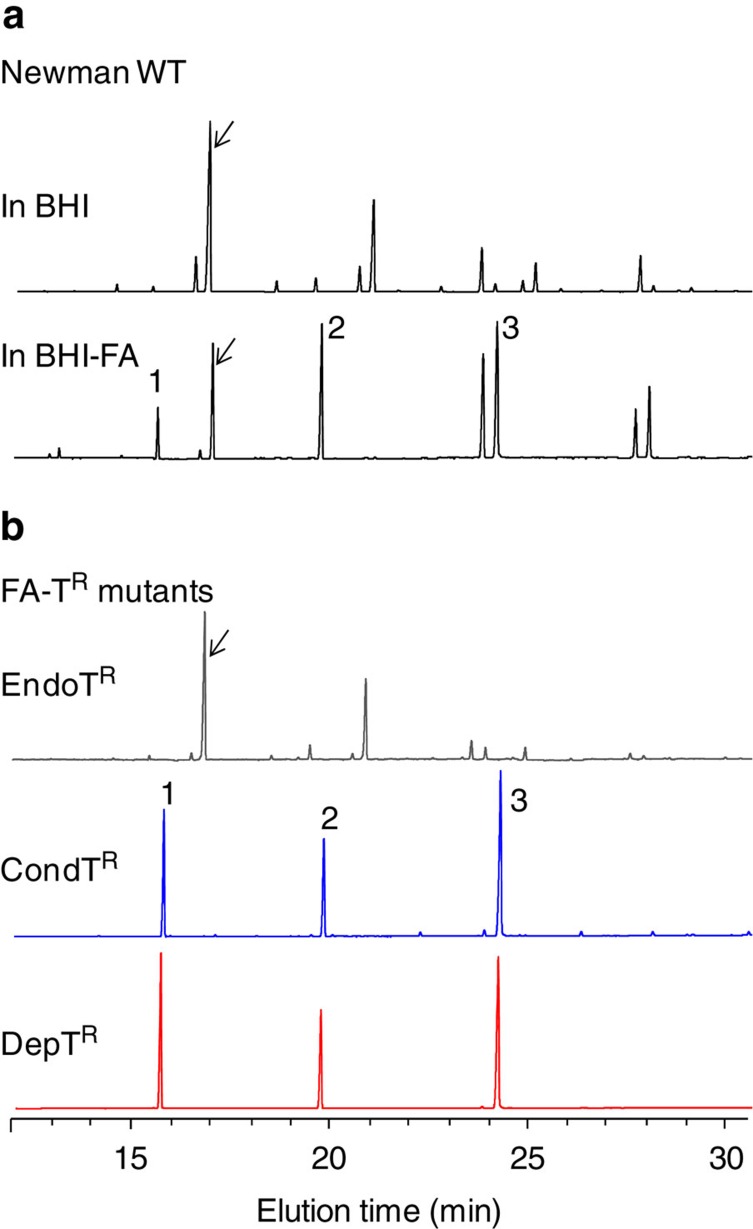
*S. aureus* Newman WT and FA-T^R^ mutants use exogenous fatty acids. Fatty acid profiles are shown for (**a**) Newman strain (WT) cultures grown in BHI and BHI-FA and (**b**) FA-T^R^ clones grown in BHI-FA-T. *S. aureus* synthesizes branched chain fatty acids *de novo*, mainly C15:0 (*ai*15:0; indicated by arrow) and its elongation products, and saturated straight chain fatty acids. In BHI-FA, the WT strain uses exogenous fatty acids, but continues *de novo* synthesis. Peaks 1, 2, and 3 correspond to the exogenous fatty acids added during growth, respectively C14:0, C16:0, and C18:1 (collectively referred to as ‘FA', see Methods). EndoT^R^, FA-T^R^ with endogenous fatty acids (grey); CondT^R^, FA-T^R^ with conditional FASII activity (blue), that is, active in BHI but bypassed in fatty acids plus triclosan; DepT^R^, FA-T^R^ requiring exogenous fatty acids for growth (red). The fatty acid profile of the EndoT^R^ mutant was similar to that of the WT in BHI, while CondT^R^ and DepT^R^ profiles are exogenous.

Fatty acid profiles of randomly selected FA-T^R^ mutants (16 prototrophs and 20 DepT^R^ mutants) were determined from whole cell extracts of cultures prepared in BHI-FA-T medium. Representative profiles of each class of mutants are shown (Fig. 1). Among prototrophs, a minor class (3/16) comprised mutants with endogenous (EndoT^R^) fatty acid profiles, indicating that the FASII pathway was active. The dominant class of prototrophs (13/16) comprised mutants having media-dependent fatty acid profiles, that is, with exogenous fatty acids in BHI-FA-T, and endogenous fatty acids in BHI (conditional triclosan-resistant mutants, referred to as CondT^R^). Mutants of this class have not previously been reported. All the DepT^R^ clones showed poor or no growth on BHI plates, but displayed exogenous profiles in BHI-FA-T medium. These results show that the majority of isolated FA-T^R^ mutants were able to bypass a FASII block by incorporating exogenous fatty acids.

### Gain of function in an *S. aureus* conditional mutant

**Figure 2:**
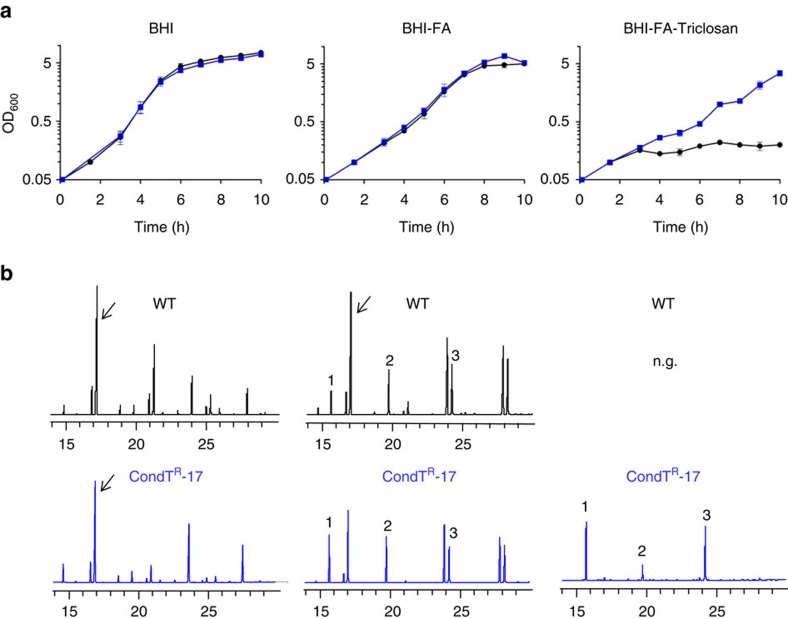
Gain of function of the *S. aureus* CondT^R^-17 mutant. (**a**) Growth of WT and CondT^R^-17 (*fabD*^*G196R*^) *S. aureus* strains in BHI, BHI-FA and BHI-FA-T media. Results show the average and range of biologically independent duplicates. (**b**) Fatty acid profiles of one sample set, taken at the 10 h time point. Endogenous branched-chain fatty acid (*ai*15:0) is indicated with arrows. Fatty acids are: 1- C14:0; 2- C16:0 and 3- C18:1, the fatty acids comprised in BHI-FA-T medium. N.g., no growth. Black, WT; blue, CondT^R^-17.

Growth and fatty acid profiles of CondT^R^-17 and the Newman strain were compared in the absence or presence of fatty acids and triclosan (Fig. 2a). Growth of both strains was comparable in BHI medium. The addition of fatty acids expectedly slowed strain growth, which was also comparable for both strains (Fig. 2a, centre)^37^,^38^. When triclosan was added, CondT^R^-17 maintained growth, but not the Newman strain. Fatty acid profiles confirmed CondT^R^ strain capacity to bypass FASII inhibition and shift to the exogenous FA source (Fig. 2b). Thus, this conditional mutant can use exogenous fatty acids upon FASII block, while displaying normal growth otherwise.

### Numerous FASII-bypass mutations map to *fabD*

Known *S. aureus* triclosan-resistant mutants carry mutations in the gene encoding FabI, the triclosan target: such mutant enzymes fail to bind triclosan but remain enzymatically active^12^,^14^,^16^,^24^. Our FA-T^R^ clones with EndoT^R^ fatty acid profiles resembled previously described strains carrying mutations in *fabI*^14^,^16^. The role of fatty acids in obtaining such mutants is unknown. In contrast, all tested mutants with exogenous fatty acid profiles (DepT^R^ and CondT^R^ strains) carried wild type *fabI* genes (Table 1).

Previously isolated *S. aureus* fatty acid auxotrophs mapped to FASII initiation genes *accC* and *accD*, encoding acetyl-CoA carboxylase subunits (biotin carboxylase and β-carboxytransferase respectively) for malonyl-CoA synthesis^27^ (Supplementary Fig. 1). Among five tested DepT^R^ mutants, one (DepT^R^-28) carried an *accB* mutation (Table 1).

Whole genome sequencing of one CondT^R^ and three DepT^R^ mutants revealed that these mutants carried *fabD* mutations as compared with the Newman parental strain (Table 1, sequenced strains are designated with ‘#'). CondT^R^-17 bore a single nucleotide substitution generating FabD^G196R^; DepT^R^-5 carried a single nucleotide insertion that truncated FabD at amino acid position 8. No other mutation was detected in either strain. Strains DepT^R^-45 and DepT^R^-72 displayed single nucleotide deletions in *fabD*, and each carried a second mutated allele (Table 1), which mapped to either NWMN_0043 encoding conserved Staphylococcal Antigen 1b (Csa1b), one of a conserved family of 18 protein homologues^39^ (for DepT^R^-45), or NWMN_1735, encoding a DNA-repair-related protein^40^ (for DepT^R^-72). We chose to focus here on the singly mutated *fabD* strains. Sequencing *fabD* of other FA-T^R^ CondT^R^ clones revealed mutations mainly at position G196, and one at position Q265 (Table 1). FabD G196 is adjacent to key residues (positions 198 and 199) of the FabD active site based on *S. aureus* FabD crystallography^41^; it is possible that substitution by less flexible amino acids than glycine might impose constraints on FabD activity. The Q265P mutation is within a conserved region separating a helix from a β-strand as suggested from *Escherichia coli* FabD structure^42^.

Thus, the majority of FA-T^R^ *S. aureus* mutants identified here contain mutations in *fabD* and utilize exogenous fatty acids to overcome FASII inhibition. Both *fabD* and *acc* intervene in FASII initiation (Supplementary Fig. 1), suggesting the importance of this first step in FASII bypass^27^.

### Diverse fatty acid combinations allow FASII bypass

**Figure 3:**
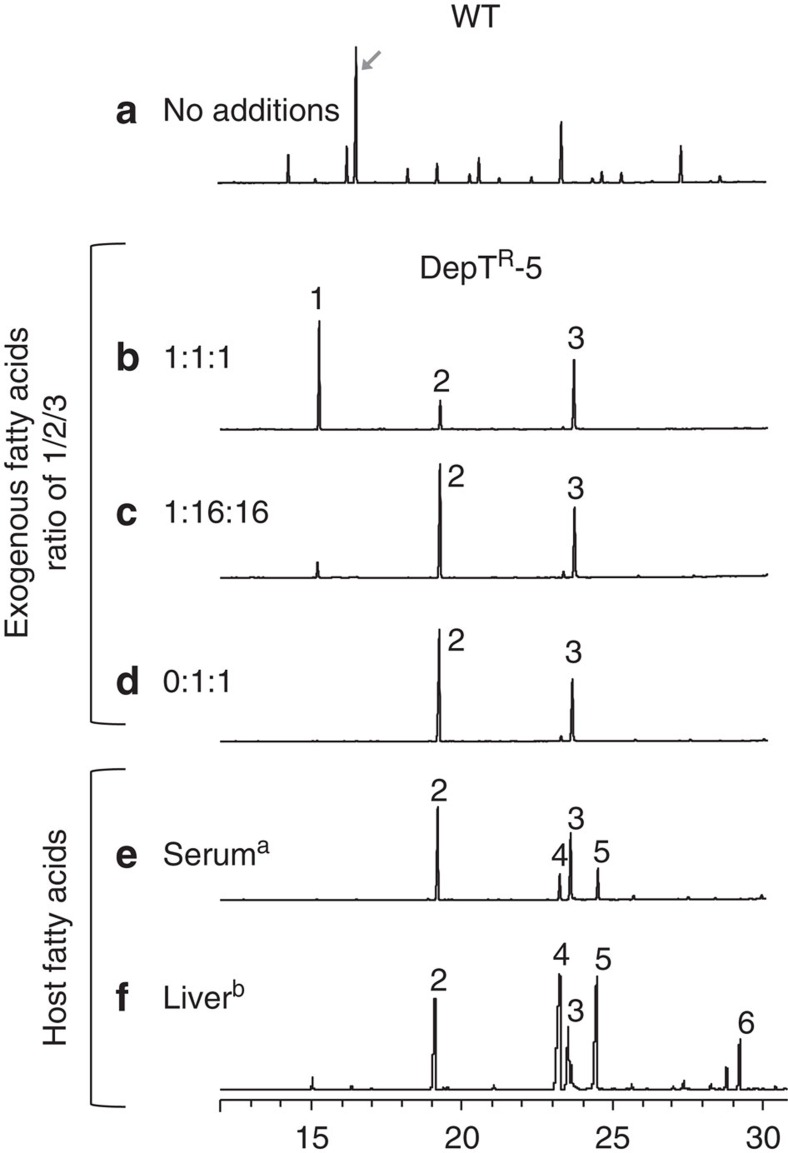
A *S. aureus fabD* mutant uses diverse fatty acid combinations for FASII bypass. (**a**) Newman strain in BHI. (**b**–**f**) Cultures of DepT^R^-5 were grown in BHI medium supplemented with triclosan and different fatty acid combinations and ratios, as indicated. Fatty acid profiles and OD_600_ were determined on cells harvested from 7.5 h (**a**–**e**) and 4.5 h (**f**) cultures. **a**, medium was supplemented with 10% delipidated serum, which lowered toxicity. **b**, Liver extract, prepared by sterile filtration, was added at a final concentration of 3% (without added fatty acids). The diagonal arrow indicates the position of *ai*15:0, the major fatty acid synthesized by *S. aureus*. Fatty acids are: 1- C14:0; 2- C16:0; 3- C18:1; 4- C18:0; 5- C18:2; 6- C20:4. Proportions of input fatty acids present in supplemented media are indicated at left. Fatty acid profile of liver extract is in Supplementary Fig. 4.

Previous studies suggested that *ai*15:0 was strictly required at the second acyl position of the *S. aureus* phospholipid^24^,^27^,^32^,^43^. However, our results indicated that other fatty acids can replace *ai*15:0. We explored the possibility that a fatty acid of similar length, like C14:0 (33% of our FA cocktail), might substitute for *ai*15:0 when FASII was blocked (Fig. 3). The DepT^R^-5 mutant was grown in medium containing triclosan and fatty acid cocktails with 33, 3 or 0% C14:0. For each cocktail, the amounts of C14:0 in *S. aureus* profiles reflected its proportions among fatty acids in the medium, indicating that C14:0 is not preferentially incorporated for FASII bypass.

A mix of the four principal fatty acids in human serum in physiological proportions (C16:0, C18:0, C18:1 and C18:2) was also tested. When added in purified form, the free fatty acid cocktail was toxic to staphylococci, likely due to C18:2 (linoleic acid), which makes up ∼35% of serum fatty acids^35^. However, growth was restored when 10% delipidated serum was added, and DepT^R^-5 displayed an exogenous fatty acid profile (Fig. 3e).

To mimic the within-host environment during infection, we grew *S. aureus* wild type and DepT^R^-5 strains in medium containing sterile-filtered pig liver and kidney extracts. The incorporated fatty acids reflected those present in the medium (shown for liver; Fig. 3f, see Supplementary Fig. 4 for organ extract profile). Unexpectedly, long-chain unsaturated fatty acids C18:2 and C20:4 were efficiently incorporated. C16:0 was the shortest abundant fatty acid incorporated, indicating that DepT^R^-5 *fabD* mutant growth was not dependent on *ai*15:0 or other short-chain fatty acids.

Thus, the *S. aureus fabD* mutant can use diverse fatty acids, including those available in the host, and can dispense with branched chain fatty acids. These results indicate that the composition of the *S. aureus* membranes can be more flexible than generally supposed^24^,^27^,^32^,^43^.

### Role of the *S. aureus fabD* allele in FASII bypass

**Figure 4:**
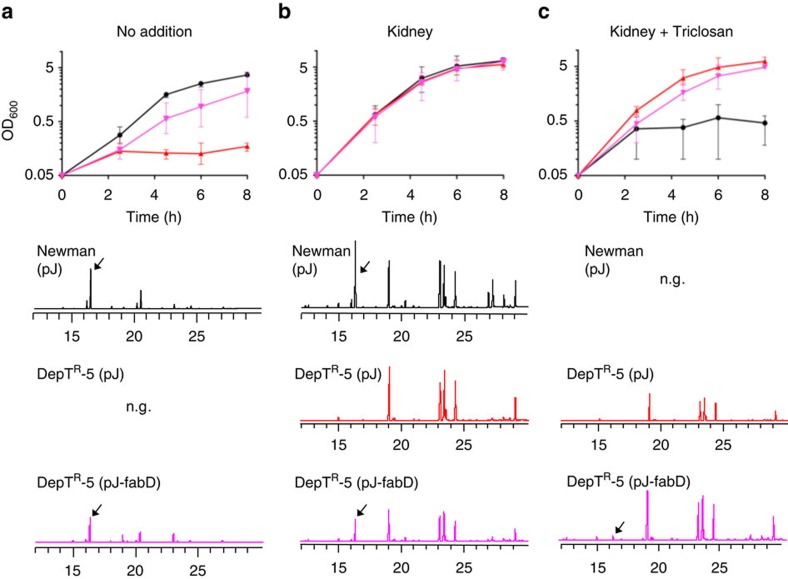
Complementation of DepT^R^-5 by the wild type *fabD* allele. Overnight LB-FA cultures of WT and DepT^R^-5 strains carrying pJ (control plasmid), or pJ-fabD (expressing the *fabD* gene) were washed twice in LB, and cultures were inoculated at a starting OD_600_=0.05 in (**a**) LB (Cm 3 μg ml^−1^ and ATc 50 ng ml^−1^), (**b**) the same medium containing sterile-filtered pig kidney extract (3% final volume) without or (**c**) with triclosan (0.5 μg ml^−1^). OD_600_ was followed over 8 h. Fatty acid profiles were determined from harvested cells, as shown below the corresponding growth condition. N.g., no growth. Reference fatty acid profile and peak identification of kidney extract is in Supplementary Fig. 4. Growth results show the average with standard deviation of three experiments that gave comparable results (Supplementary Fig. 5). However, despite precautions, growth was variable between independent experiments, which may be due to the counter-selective effect of triclosan on the pJ-fabD plasmid.

To confirm that the *fabD* mutation is responsible for fatty acid dependency of DepT^R^-5, the wild type *fabD* gene was cloned in expression vector pJ (resulting in pJ-fabD) and established in the mutant strain background. To simulate *in vivo* conditions and obtain insight into *S. aureus fabD* mutant behaviour in the animal host, tests were performed in medium supplemented with liver and kidney extracts as fatty acid sources. In non-supplemented medium, the pJ-fabD plasmid restored *ai*15:0 synthesis and growth to the DepT^R^-5 strain, confirming that the *fabD* mutation was responsible for the FASII defect (Fig. 4a). Importantly, growth of DepT^R^-5 (pJ) in host organ extracts was comparable to that of the Newman (pJ) strain, indicating that DepT^R^-5 can grow robustly while using exclusively host fatty acids. In this condition, complementation with pJ-fabD also resulted in *ai*15:0 synthesis (Fig. 4b). In triclosan plus host organ extracts, growth of DepT^R^-5 (pJ-fabD) lagged slightly behind that of DepT^R^-5 (pJ) (Fig. 4c, Supplementary Fig. 5a). The intermediate effect could indicate that triclosan exerts a counter-selective effect on the plasmid-carried *fabD* copy.

CondT^R^-17 grew both without and with fatty acid supplementation, and as expected, strong phenotypic differences were not observed in CondT^R^-17 complementation tests. The CondT^R^-17 (pJ or pJ-fabD) strains grew like Newman (pJ) in host organ extracts (data not shown). However, as above, CondT^R^-17 (pJ-fabD) displayed a slight growth retardation compared with CondT^R^-17 (pJ) in fatty-acid-triclosan-containing media as shown in the presence of kidney extracts (Supplementary Fig. 5b). In view of the absence of other mutations in the CondT^R^-17 strain, the identification of other ‘CondT^R^' isolates mutated in *fabD* (Table 1), and the existence of *fabD* variants with FA-T^R^ phenotypes among clinical isolates (below), we consider that *fabD*^*G196R*^ is responsible for the CondT^R^-17 conditional phenotype.

These results lead us to conclude that the *fabD* allele is responsible for the FASII defect in DepT^R^-5 and CondT^R^-17 strains. Both mutants grew comparably to the WT strain in host-simulated conditions, suggesting that these mutants would behave like the wild type strain in host compartments.

### Cross-resistance of *S. aureus fabD* mutants to FASII inhibitors

**Figure 5:**
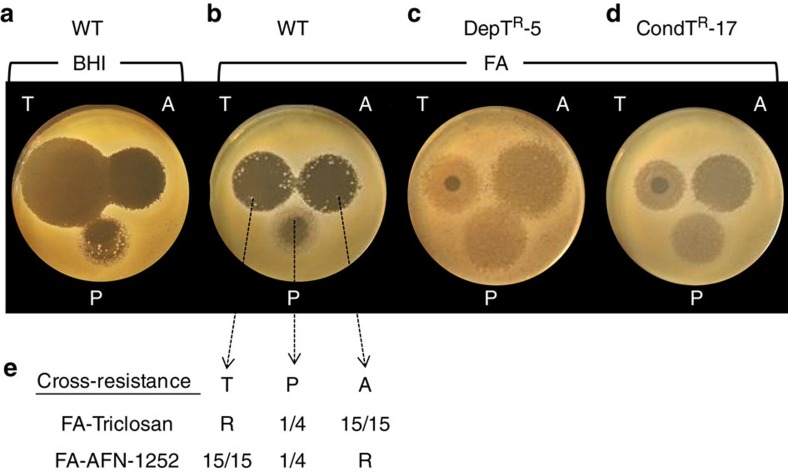
*S. aureus* cross-resistance to anti-FASII drugs in the presence of exogenous fatty acids. Overnight BHI-FA-grown cultures of Newman WT, DepT^R^-5, and CondT^R^-17 were washed twice in BHI and plated on BHI solid medium without (**a**) (for Newman strain) or with fatty acids (**b**–**d**). Plates were spotted with triclosan (‘T', 1.5 μg), AFN-1252 (‘A', 1 μg), and platensimycin (‘P', 8 μg), respectively 100, 100 and 16 times the MIC. Photographs were taken after 48 h at 37 °C. (**c**) DepT^R^-5 and (**d**) CondT^R^-17 strains accrue resistance to all three drugs on FA plates as seen by growth within the halo region, as compared with (**b**) the WT strain. The small inhibitory zone around triclosan is attributed to non-specific membrane effects^12^. (**e**) colonies that appeared within the inhibitory zones of WT strains on BHI-FA plates were cross-patched on BHI, BHI-FA, BHI-FA-AFN-1252 (80 ng ml^−1^) and FA-triclosan (250 ng ml^−1^). All colonies picked in fatty acids as triclosan-resistant were AFN-1252-resistant, and conversely. Fatty acid profiles (not shown) determined for colonies from each anti-FASII selective plate were fully exogenous.

AFN-1252 is a FabI inhibitor in clinical trials^44^; platensimycin is a FabF inhibitor and antimicrobial candidate^11^. Cross-resistance between triclosan and these drugs was tested. *S. aureus* Newman (WT) and *fabD* mutants DepT^R^-5 and CondT^R^-17 were plated on BHI or BHI-FA media. FabI inhibitors triclosan and AFN-1252, and the FabF inhibitor platensimycin were spotted on lawns (at 100, 100 and 16 times the respective minimal inhibitory concentrations (MIC)) (Fig. 5a–d). Triclosan- and AFN-1252- resistant colonies appeared within the inhibitory zones of the WT strain only on fatty-acid-containing plates (compare Fig. 5a,b). Platensimycin was poorly effective in both media at the tested concentration. We also noted a pronounced decrease in the inhibitory zone diameter for triclosan on BHI-FA compared with BHI plates (compare Fig. 5a,b). In contrast, both DepT^R^-5 and CondT^R^-17 strains grew, albeit more slowly, within inhibitory zones of the 3 FASII inhibitors on BHI-FA plates (Fig. 5c,d).

Cross-resistance was then checked for colonies isolated from WT BHI-FA plates as triclosan, AFN-1252 or platensimycin resistant (Fig. 5b). Colonies were patched on BHI-FA medium containing triclosan (0.25 μg ml^−1^) or AFN-1252 (0.08 μg ml^−1^) (Fig. 5e). Fatty acid profiles of colonies as determined on primary selections and cross-selections were all exogenous (not shown). In addition, liquid cultures of DepT^R^-5 and CondT^R^-17 confirmed the capacity of DepT^R^-5 and CondT^R^-17 to grow on platensimycin and incorporate exogenous fatty acids (Supplementary Fig. 6).

These results further support a role of fatty acids in facilitating *S. aureus* escape from anti-FASII inhibition, and show that mutants selected by the use of a FASII inhibitor can be resistant to other FASII inhibitors.

### Infection by *S. aureus fabD* mutants

**Figure 6:**
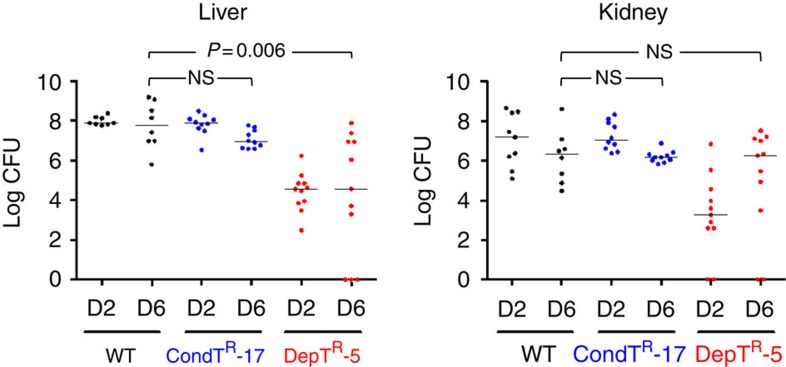
Infection and persistence of *fabD* isolates in a mouse model. Newman WT (black), CondT^R^-17 (*fabD* conditional; blue), and DepT^R^-5 (*fabD* fatty-acid-dependent; red) strains were prepared. 10^6^ CFU were injected in the tail veins of 6-week female BALB/c mice (in groups of 8–11). In these experiments, one WT-infected and one CondT^R^-17-infected mouse died 4 days post-infection. At days 2 (D2) and 6 (D6), animals from each group were sacrificed and dissected. Liver and kidney were homogenized by ultra-turrax and CFU per organ were determined by serial dilutions on BHI-FA plates containing 10% serum. Results are pooled from two independent experiments. Statistical tests were done using Mann–Whitney U to determine differences in CFU between groups on D2 and/or D6. No significant differences were observed between WT and CondT^R^-17 in any test condition. Notably, no significant difference was observed between WT and DepT^R^-5 CFUs in kidneys at D6 (*P*=0.77). NS, non-significant differences. The detection limit was 10^2^ CFU per organ; samples with no CFUs were plotted as ‘0'. In DepT^R^-5 samples, one mouse showed no detectable counts in liver or kidney.

Infections by *fabD* DepT^R^-5, *fabD* CondT^R^-17 or the WT strain were compared in a mouse sepsis model. A low inoculum (10^6^ CFU) was used to avoid the possibility of false-positive virulence assignments^32^. The conditional FA-T^R^ mutant, CondT^R^-17, was as infective as the parental Newman strain and gave equivalent CFUs in both liver and kidneys (Fig. 6). These results indicate that this strain, and possibly similar variants, can retain full virulence.

The DepT^R^-5 mutant showed delayed kinetics of infection. At two days post-infection, significantly lower CFUs were present in both liver and kidneys compared with the WT strain (*P*=0.001 in both organs using the Mann–Whitney *U*-test). However, these differences narrowed at 6 days post-infection, particularly in kidneys, where DepT^R^-5 CFUs increased and were not statistically different from WT CFUs (*P*=0.77 in kidneys) (Fig. 6). Colonies issued from the 6-day infection were confirmed to maintain fatty acid growth dependency, and two colonies selected at random from each organ were confirmed to carry the original *fabD* mutation.

Our results indicate that both CondT^R^ and DepT^R^ mutants would resist challenge by a FASII inhibitor *in vivo*. As DepT^R^-5 and CondT^R^-17 also proliferate and incorporate fatty acids in the presence of AFN-1252 and platensimycin (Fig. 5c,d), we speculate that both types of mutants might override FASII inhibition regardless of the FASII inhibitor.

### FASII bypass in natural isolates carrying FabD polymorphisms

Our results show that the CondT^R^-17 G196R substitution correlates with FASII bypass *via* incorporation of exogenous fatty acids. We searched public databases for natural *S. aureus* isolates carrying FabD modifications at the G196 position, and found three such isolates. Two independent sputum and nare isolates carry FabD^R196^: M0251 (a methicillin-resistant *S. aureus* (MRSA) clonal complex CC5 isolate, BioSample accession code SAMN02325289) and M1532 (BioSample accession code SAMN02364048). The third strain, SA40, is a community- and epidemic-associated methicillin-resistant sequence type ST59 that was isolated from a healthy child and carries a FabD^S196^ protein. A closely related ST59 strain SA957 is an MRSA blood isolate that does not carry the FabD^196^ polymorphism^45^. These *S. aureus* isolates as well as two FAT^R^ mutants were tested for their capacity to bypass FASII. The three strains carrying FabD^196^ polymorphisms, but not the Newman or SA957 strains, showed FASII bypass on BHI-FA-T liquid medium, as verified by growth and exogenous FA incorporation (Table 2). These results further support the correlation between *fabD*^*196*^ polymorphism and anti-FASII resistance, and show the capacity of *S. aureus* human isolates to use exogenous fatty acids to overcome FASII inhibitors. They also indicate that anti-FASII-resistance is present in some commensal and clinical isolates.

### Redistribution of acyl-ACP pools in *fabD* variants

**Figure 7:**
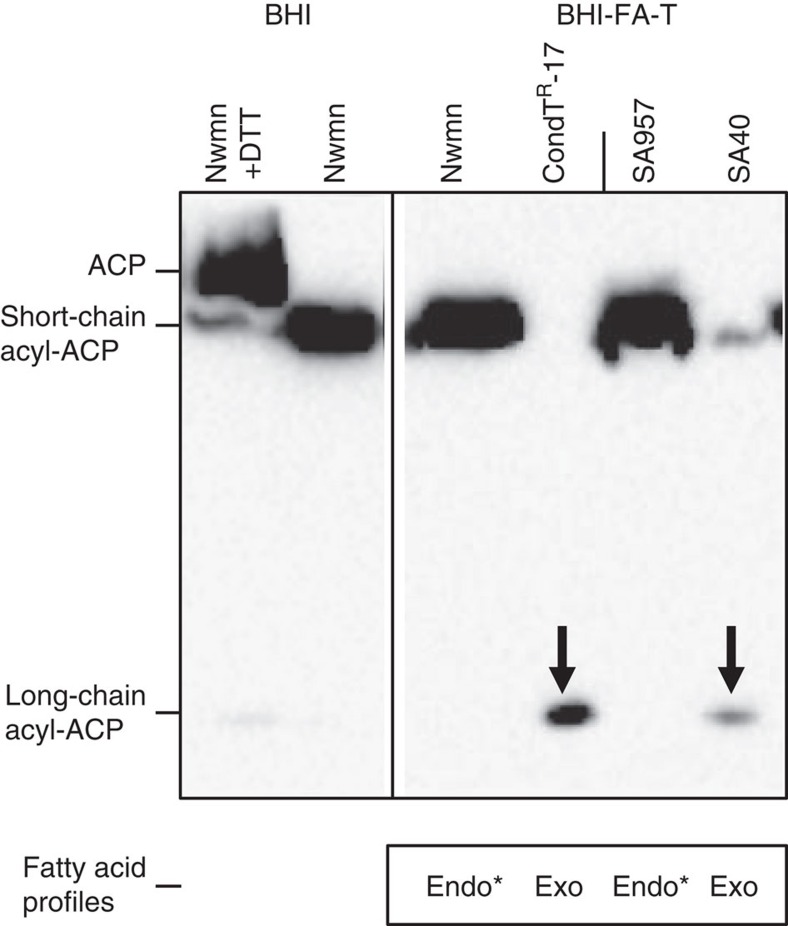
Shift in ACP species in strains with *fabD*^*196*^ polymorphisms. The impact of an altered amino acid at FabD position 196 on acyl-ACP pools was tested by comparing strain pairs: Newman and CondT^R^-17 (G196 and R196 respectively), and the ST59 pair SA957 (virulent) and SA40 (nasal isolate)^45^ (G196 and S196 respectively). Extracts prepared from 4 h cultures in BHI-FA-T medium were run on conformation-sensitive gels. Anti-ACP antibodies were used to identify ACP moieties after gel transfer. In these gels, migration of acyl-ACP species increases with longer acyl chain length^46^,^66^. DTT treatment reduces acyl-ACP thioesters and releases free ACP^47^; the DTT-treated Newman protein extract from BHI-grown cells was used as migration control. Short-chain acyl-ACP is predominant in reference strains, and long-chain acyl-ACP moieties (arrows) are detected only in extracts of *fabD* variants. Results of fatty acid profiles of reference strains and *fabD* variants are indicated below respective lanes. Nwmn, Newman strain; *, fatty acid profiles derived from non-growing cultures. The Western is from a single gel with the same exposure, and results are representative of 3 biologically independent experiments. The Western blot shown above corresponds to the entire gel.

FabD catalyses the transfer of malonyl from malonyl-CoA to acyl-carrier proteins (ACPs), yielding malonyl-ACP (Supplementary Fig. 1). We considered that FabD polymorphisms near the active site (that is, position 196) might lower the efficiency of this reaction, thus leaving free ACP available for exogenous fatty acid incorporation. Two strain pairs were used to test this hypothesis: Newman strain (*fabD*^*G196*^)–CondT^R^-17 (*fabD*^*R196*^) and ST59 clinical strains SA957 (*fabD*^*G196*^)–SA40 (*fabD*^*S196*^). Cell extracts and culture supernatants were prepared from 4 h cultures in BHI-FA-T. Short- and long-chain acyl-ACP were assessed on Western blots of conformation-sensitive gels using anti-ACP antibodies^27^,^46^; fatty acid profiles of cell extracts were done in parallel (Fig. 7). A dithiothreitol (DTT)-treated extract of BHI-grown Newman strain was used to identify the migration position of free ACP^27^,^46^,^47^.

Acyl-ACP species were redistributed in both *fabD* variants (CondT^R^-17 and SA40) compared with respective reference strains. Short-chain acyl-ACP pools were decreased in both variant strains, which is consistent with a reduced FabD activity as hypothesized. In addition, the *fabD* mutants contain long-chain acyl-ACPs that are not detected in reference strains, and their fatty acid profiles (but not those of the reference strains) are fully exogenous. Certain fatty acids are known to induce ACP release from *S. aureus* cells^38^; however, we found that at 4 h, supernatants of all strains produced weak signals that did not differ significantly between strain pairs (Supplementary Fig. 7). Thus, we hypothesize that the FabD^R196^ and FabD^S196^ mutant enzymes may be less active than FabD^G196^, allowing free ACPs to be loaded with exogenous long-chain fatty acids that can then be incorporated into membrane phospholipids.

## Discussion

The present work shows that a fatty-acid-rich environment can favour the emergence of *S. aureus* strains displaying resistance to multiple FASII inhibitors. Point mutations in *fabD* appear to facilitate FASII bypass by enhancing incorporation of exogenous fatty acids. Conditional (CondT^R^) *fabD* mutants can grow normally, and can also bypass FASII in the presence of exogenous fatty acids, which suggests that they might be able to spread successfully in environments such as host organs. The *S. aureus fabD* mutants can use various fatty acid combinations (Fig. 3). This flexibility indicates that, contrary to previous assumptions^24^,^27^,^43^, the branched-chain fatty acid requirement in *S. aureus* is not strict, as shown here for *fabD*^*196*^ variants in laboratory and clinical strains from major lineages (CC8, CC5 and ST59). It may also help survival of anti-FASII-resistant bacteria in different host compartments that vary in fatty acid composition. Therefore, we propose that all these factors need to be considered when developing anti-FASII drugs for treating *S. aureus* infection^4^,^5^,^8^,^10^,^11^,^48^,^49^.

**Figure 8:**
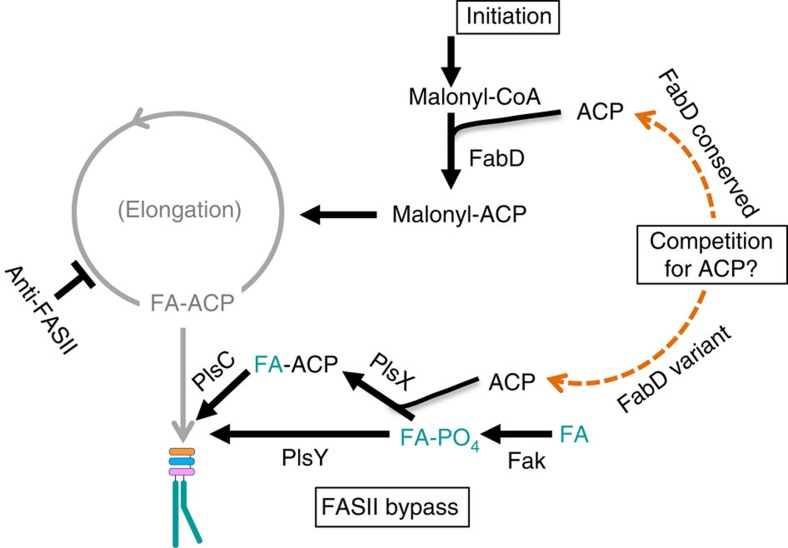
Model of FASII bypass by *S. aureus fabD*^*196*^ variants based on competition for ACP. A role for FabD in ACP balance and as a determining factor of FASII bypass is proposed. Indicated pathways and regulatory functions are as previously identified^43^,^67^. In grey, the inhibited FASII elongation pathway. FASII initiation enzyme FabD uses ACP and malonyl-CoA to synthesize malonyl-ACP^50^. In FASII inhibitory conditions, PlsX would compete for ACP pools to convert exogenous fatty acids (turquoise) into phospholipids. Less efficient activity of FabD^196^ variants (Fig. 7) would leave ACP available for the PlsX reaction in the indicated direction (PlsX is a potentially reversible enzyme^43^), as indicated by orange dashed arrow. Fak-PlsY and Fak-PlsX-PlsC as proposed here, mediate acyl group joining respectively to positions 1 and 2 of the glycerol-3-phosphate (G3P) backbone^31^,^43^. FA, fatty acid; FA-ACP, acyl-ACP; FA-PO_4_, acyl-phosphate.

In FASII-bypass conditions, *fabD*^*196*^ variant strains show decreased levels of short-chain acyl-ACPs and increased levels of long-chain acyl-ACPs, in comparison with reference strains (Fig. 7). The decrease in short-chain acyl-ACP is consistent with our proposal that FabD^196^ variants may have reduced enzyme activity. We speculate that the detected long-chain acyl-ACP species correspond to exogenous fatty-acyl-ACP intermediates in phospholipid synthesis, which is consistent with the exogenous fatty acid profiles of *fabD*^*196*^ variants (Fig. 7). FASII bypass might depend on the outcome of competition for ACP between two enzymes at opposing ends of the pathway: the FASII initiation enzyme FabD (which uses ACP to synthesize malonyl-ACP) and the post-FASII termination enzyme PlsX (which catalyses the reversible formation of acyl-phosphate from acyl-ACP)^43^,^50^. Lower FabD activity would leave more ACP available for PlsX, to drive the reaction towards the synthesis of long-chain acyl-ACP (the phospholipid synthesis precursor) from exogenous fatty acids, and thus lead to FASII bypass (Fig. 8). At present, this or other roles for long-chain acyl-ACP, for example, in modulating FASII enzyme activities, need to be demonstrated.

Previous results showing that fatty acid auxotrophs carrying mutations in *acc* genes are avirulent led to the hypothesis that FASII-bypass mutants would not survive in the host^27^,^32^. In contrast, we have shown here that the *fabD* mutants DepT^R^-5 and CondT^R^-17 are infectious and persist in the host, and that clinical isolates chosen as bearing FabD^196^ polymorphisms can bypass FASII inhibition (Fig. 6 and Table 2). We speculate that this apparent discrepancy may be due to the different effects of the *acc* and *fabD* mutations on malonyl-ACP pools. FapR is a transcriptional repressor of FASII genes and of phospholipid synthesis genes *plsX* and *plsC*^51^ (Supplementary Fig. 1). FapR activity is alleviated by malonyl-CoA^51^,^52^,^53^. Mutations in *acc* leading to reduced synthesis of malonyl-CoA would result in FapR-mediated repression of phospholipid synthesis. On the contrary, malonyl-CoA would not be affected in the *fabD* mutants; it is even possible that the *fabD* mutants may accumulate malonyl-CoA, thus alleviating FapR-mediated repression of phospholipid synthesis genes. This situation would favour FASII bypass in environments rich in fatty acids.

The present study uncovered *fabD* mutants as conferring a potentially non-deleterious anti-FASII escape strategy. It is possible that *fabD* mutations were not identified previously because of differences in experimental conditions, for example, in the choice of fatty acids and/or FASII inhibitors used in selections. Alternatively, strains might differ in their capacity to mediate FASII-bypass *via* mutations. Nevertheless, identification of *fabD* variant strains belonging to CC5, CC8, and ST59 groups and including MRSA isolates suggests that FASII-bypass variants may emerge in these dominant lineages.

Our study revealed that FASII bypass mutants need not be fatty-acid auxotrophs. The CondT^R^ mutant is resistant to several FASII inhibitors, yet grows normally and is infectious, indicating a gain of function. CondT^R^-like variants may thus present a risk of dissemination in the environment. The three tested clinical and commensal isolates with *fabD*^*196*^ polymorphisms were all anti-FASII resistant (Table 2). Among them, differential anti-FASII resistance of closely related ST59 strains SA40 (resistant) and SA957 (sensitive) that differ in *fabD*^*196*^ further supports a role for *fabD* in FASII bypass among natural isolates. As FabD ORFs in genome-sequenced isolates are highly conserved within clonal types, we speculate that polymorphisms associated with FASII bypass arose by *in vivo* selection, possibly due to intensive use of triclosan in hygiene and household products^12^. A recent study of nasal secretions correlated the presence of triclosan with a greater incidence of colonizing *S. aureus* and consequent infection risk^18^. Interestingly, nasal secretions are fatty-acid-rich^54^, and the presence of triclosan or other FASII inhibitors could select for high frequency FASII bypass. The CondT^R^-like human nasal MRSA isolate (SA40) may be a case in point.

DepT^R^-like mutants are also likely to be relevant to infection. Results presented here indicate that DepT^R^-5 persists in the kidney over a six-day infection period (Fig. 6), indicating that these bacteria survive in the infected host. A recent study identified a FASII defect among clinical *S. aureus* small colony variants (SCV). SCV growth was restored to normal by fatty acid supplementation^55^, strongly pointing to a DepT^R^-like phenotype and implicating FASII bypass in chronic infection. Similar findings were reported for the opportunist pathogen *Enterococcus faecalis*^56^. Abscess formation during *S. aureus* infection is common, and it is tempting to speculate that this stage of infection may give privileged access to host fatty acids that support growth of both CondT^R^ and DepT^R^ mutants.

Fatty-acid-rich skin^36^,^57^, possibly enriched by fatty-acid-containing skin products^58^, may be an ideal medium on which FASII inhibitors would select for FASII-bypass-competent staphylococci. The selection strategy used in our study mimicked this situation. Emergence of versatile *S. aureus* FASII bypass variants upon anti-FASII treatment may generate a long-term risk for persistence and infection^59^, requiring new rounds of antimicrobial therapy.

## Methods

### Bacterial strains and growth conditions

Experiments were performed using the *S. aureus* Newman strain^60^. SA40 (an Asian-Pacific nasal ST59 MRSA isolate from a healthy child) and SA957 (a virulent Taiwan ST59 blood isolate) were kindly provided by Freidrich Götz and Regine Stemmler (U. Tübingen, Germany)^45^. The M0251 (MRSA, clonal complex CC5) and M1532 strains are respectively nasal and sputum isolates that were generously provided by Mary Delaney and Michael Calderwood, and are part of the Brigham and Women's Hospital collection (Boston, USA). Plasmid cloning and extraction were performed in *E. coli* strains Top10 (Invitrogen, France) and MG1655 (ref. 61). BHI and LB media were used for *S. aureus*, and LB for *E. coli* growth. The fatty-acid mixture (referred to as ‘FA') comprises C14:0 (myristic acid), C16:0 (palmitic acid), and C18:1 (oleic acid); 100 mM stocks in dimethylsulphoxide (DMSO) of each fatty acid were mixed at a 1:1:1 ratio, and then diluted 1:200 for use in liquid or solid medium. Other fatty acid mixtures were prepared similarly, such that the final molar concentration of fatty acids was 0.5 mM (that is, 0.17 mM each fatty acid). All fatty acids were from Larodan Fine Chemicals (Sweden). Newborn calf serum (Sigma-Aldrich, France) and delipidated calf serum (Eurobio, France) were added to growth medium (10% final concentration) as indicated. Triclosan (Irgasan; Sigma-Aldrich) was routinely used at 0.25 μg per mL in liquid cultures; this is 15 times the MIC for *S. aureus* Newman strain in BHI medium. Platensimycin (Tebu-Bio, France) and AFN-1252 (MedChem Express) were used respectively at 2 μg ml^−1^ and 80 ng ml^−1^ as per published efficacies^11^,^27^. Anti-FASII drugs were prepared in DMSO.

Kidney and liver extracts from adult pigs (usually 6 months, purchased at a local butcher shop) were prepared as follows: 200 g of each organ were cut in 0.5 cm cubes, and resuspended in PBS (1:2 w-v). Samples were homogenized using an ultra-turrax and then centrifuged twice (4,000 g for 30 min) to remove solid material. Supernatants were filtered through Whatman paper, recentrifuged at 20,000 g, and then sterile-filtered sequentially on 0.45 and 0.2 micron membranes. All steps were performed at 4 °C, and extracts were stored at −20 °C.

### Selection for triclosan resistance on FA plates

Dilutions of overnight *S. aureus* Newman cultures were plated on four types of medium: non-selective (BHI); BHI plus FA (BHI-FA; FA are as described above); BHI with triclosan (BHI-T (0.25 μg ml^−1^)), BHI containing FA and triclosan (BHI-FA-T (0.25 μg ml^−1^)). Plates were incubated at 37 °C and scored at 48 h. Colonies were selected for phenotypic confirmation and characterization.

Colony forming units (CFU) on plates were compared with determine FA-T^R^ frequencies. Colonies that appeared on BHI-FA-T plates were then patched on the four plates as above to distinguish mutational phenotypes. Proportions of DepT^R^ and CondT^R^ mutants varied in independent experiments, possibly suggesting that slight variations in conditions may impact mutant phenotypes. Changes in fatty acid profiles of some FA-T^R^ isolates were observed upon FA-T selection, particularly after storage, suggesting that adaptation might occur during selection and/or storage. Care was therefore taken in analysing profiles directly from selective plates and before storage.

### Determination of fatty acid profiles

Whole cell esterified fatty acid determinations were done essentially as described^62^. Briefly, 1–2 ml *S. aureus* cultures (OD_600_=≥1) were centrifuged, washed once in 0.9% NaCl containing 0.02% Triton X-100, then washed twice in 0.9% NaCl. Cell pellets were treated with 0.5 ml of 1 N sodium methoxide. Heptane (200 μl) was then added, together with methyl-10-undecenoate (Sigma-Aldrich) as internal standard, vortexed for 1 min, and centrifuged. Fatty acid methyl esters were recovered in the heptane phase. Analyses were performed in a split-splitless injection mode on an AutoSystem XL Gas Chromatograph (Perkin-Elmer) equipped with a ZB-Wax capillary column (30 m × 0.25 mm × 0.25 μm; Phenomenex, France). Data were recorded and analysed by TotalChrom Workstation (Perkin-Elmer). *S. aureus* fatty acid peaks were detected between 12 and 30 min of elution.

### DNA sequencing

The *fabI*, *accABCD* and *fabD* genes of selected *S. aureus* Newman strain FA-T^R^ clones were sequenced. Primer pairs used for DNA amplification were: for *fabI*: FabIfd 5′-GATACAGAAAGGACTAAATCAAA-3′ and FabIrev 5′-TTTCCATCAGTCCGATTATTATA-3′ (ref. 20); for *accDA*: AccDAfd 5′-AACTAATGTATTGAATTGATGTAAACG-3′ and AccDArev 5′-AACATTCAACAGTCAAACGA-3′; for *accBC*: two sets were used AccBC1fd 5′-ACGGGTAGATGAAAACAAAC-3′ and AccBC1rev 5′-TCTTTTTCATCACGAGCAA-3′, and AccBC2fd 5′-AGTTGTTCCTGGTAGTGACG-3′ and AccBC2rev 5′-CCAGTGATGCCTTCGACTTC-3′; for *fabD*: FabDfd 5′-GAAGGTACTGTAGTTAAAGCACACG-3′ and FabDrev 5′-GCTTTGATTTCTTCGACTACTGCTT-3′.

Whole genome sequencing was done on DepT^R^ isolates DepT^R^-5, DepT^R^-72 and DepT^R^-45, CondT^R^ mutant CondT^R^-17, and our parental Newman strain. Libraries were prepared with genomic DNA using the Illumina MiSeq next generation sequencing system (IMGM, Germany). The 2 × 250 base pair reads were aligned and sequence variations were analysed using CLC Genomics Workbench 5.5.2 software (CLC Bio). SNPs were found when comparing our control Newman strain to that in the Newman reference sequence (GenBank Nucleotide accession code NC_009641). Only differences between our laboratory Newman strain and FA-T^R^ mutants were considered in genome comparisons.

### Cloning of *fabD* and *acpP*

The *fabD* gene (locus NWMN_1140) and the *fabD*^*G196R*^ variant gene were PCR-amplified from chromosomal DNA prepared from the Newman strain using the forward primer fabD-tail-F (5′-ATAAGCCGGGGGATCCACTAATGAGTAAAACAGCAATTATTTTTCCGGG -3′) and reverse primer fabD-tail-R (5′-GCGCGCAATTAACCCTCACTAAAGGGTTAGTCATTTTCATTC CATCCTTTCACATC-3′). Amplified DNA was cloned by Gibson assembly^63^. The vector, pJIM2246-ptetR^64^, was amplified for *fabD* cloning using primers pJ-fabD-R (5′-CCCGGAAAAATAATTGCTGTTTTACTCATTAGTGGATCCCCCG GCTTAT-3′) and pJ-fabD-F (5′-GATGTGAAAGGATGGAATGAAAATGACTAACCCTTTAGTGAGGGTTAATTGC GCGC-3′). Ligations were initially transformed into *E. coli* Top10. After plasmid verification by DNA sequencing, plasmids with and without inserts were transformed by electroporation into *S. aureus* strain RN4220 (ref. 60), re-extracted and transformed into Newman WT and *fabD* mutants. Standard electroporation protocols were followed^65^, except that DepT^R^-5 competent cells were prepared in BHI medium containing 10% heat-inactivated newborn calf serum and FA.

The *acpP* gene (locus NWMN_1142) was cloned in plasmid pMalC4X (New England Biolabs) as a fusion to *mbp*. Briefly, *acpP* was PCR-amplified using forward primer 5′-GCGAATTCGAAAATTTCGATAAAGTAAAAGATATC-3′ and reverse primer 5′-GGGAAGCTTTTATTTTTCAAGACTGTTAATAAATTTAAC-3′. The digested fragment was then ligated to EcoRI-HindIII-linearized pMalC4X, and cloned in *E. coli* TG1, giving plasmid pMalC4X-acpP.

### Complementation tests

Overnight cultures of wild type and *fabD* mutant strains carrying plasmids pJ or pJ-fabD were used to inoculate cultures at a starting density of OD_600_=0.05, in the following media: (i) LB containing 3 μg ml^−1^ Cm and 50 ng ml^−1^ anhydrotetracycline (ATc), (ii) same as (i) plus 10% kidney extract and (iii) same as (ii) plus 0.5 μg ml^−1^ triclosan. Growth was monitored in three independent experiments. Growth lags of DepT^R^-5 (pJ-fabD) in condition (iii) compared with DepT^R^-5 (pJ), and of CondT^R^-17 (pJ-fabD) compared with CondT^R^-17 (pJ) were determined as a ratio in individual experiments, and plotted as the ratio of the OD_600_ values at different time points (Supplementary Fig. 5).

### WT and *fabD* mutant resistance to FASII inhibitors

WT, DepT^R^-5 and CondT^R^-17 strains were grown overnight in BHI-FA, washed twice in BHI, and 100 μl of OD_600_=0.1 bacterial suspension were plated on solid BHI or BHI-FA medium to obtain a lawn. Triclosan (1.5 μg in 3 μl), AFN-1252 (1 μg in 3 μl), and platensimycin (8 μg in 4 μl) were spotted immediately after plating. Plates were incubated at 37 °C and photographed after 48 h. Colonies growing within the inhibition zone of the anti-FASII drugs were patched to BHI, or to BHI-FA media containing or not triclosan 0.25 μg ml^−1^, or AFN-1252 0.08 μg ml^−1^, and scored for growth after 48 h. Bacterial patches were harvested directly for fatty acid profile evaluation.

### Mouse infection model

Overnight cultures of DepT^R^-5, CondT^R^-17 and WT strains were grown in BHI-FA containing 10% serum. The morning of infection they were diluted to obtain a starting OD_600_=0.02 (WT and CondT^R^-17) and 0.04 (DepT^R^-5). Cells were grown in the same medium to OD_600_=1, washed, and resuspended in 0.9% NaCl to obtain 2 × 10^6^ cells per mL, as confirmed by plating on BHI-FA plus 10% serum solid medium. Six-week female BALB/c mice (Janvier Labs, France) were injected in the tail vein with 10^6^ cells in 500 μl 0.9% NaCl (8–11 animals per group). Animals were sacrificed at day 2 and day 6 (4–6 animals per time point) and dissected. Liver and kidneys were suspended in phosphate-buffered saline and homogenized with an ultra-turrax. CFUs were determined per organ type on BHI-FA plates containing 10% serum. DepT^R^-5 and CondT^R^-17 clones (20 for each mutant per organ), issued from day 6 platings, were randomly selected to verify fatty-acid-dependent and conditional phenotypes; conservation of the mutated alleles was further confirmed in two clones from each organ by PCR-sequencing. The presented data are pooled from animal studies performed as two independent experiments.

### Statistical analyses

The Mann–Whitney *U*-test was used to evaluate whether differences in CFUs in mice infected by Newman or mutant derivatives were statistically significant. Differences were compared for day 2 or day 6 between Newman and mutant derivatives. Analyses were performed with GraphPad Prism Software Version 6.00 (GraphPad Software, USA). Statistical significance was given as *P* values.

### Growth capacity of *fabD* clinical variants and mutants on FA-triclosan

Pre-cultures of Newman, M0251, M1532, SA957, SA40, CondT^R^-4, and CondT^R^-17 strains were prepared in BHI medium, and used to inoculate BHI or BHI-FA-T medium at an initial OD_600_=0.01. Cell density was determined as OD_600_ measurements after 16 h growth. FA profiles were determined for cultures that reached OD_600_ values above 0.5, which was defined as the growth threshold. Experiments were repeated three times.

### ACP assessment by conformation-sensitive gels

Anti-*S. aureus-*ACP antibodies were prepared using ACP purified from *E. coli* TG1 carrying pMalC4X-acpP. For this purpose, cultures were grown in LB ampicillin (100 μg ml^−1^), and ACP-MBP expression was induced by IPTG 0.5 mM for 2 h during mid-exponential growth. The ACP-MBP fusion was recovered on a maltose resin and eluted with 20 mM maltose. The fusion protein was digested with Xa protease as per manufacturer's instructions (New England Biolabs). Purified ACP was recovered from polyacrylamide gel electrophoresis–SDS (PAGE–SDS) gels and used for rabbit antibody preparation (Covalabs). The antibody preparation was validated on purified ACP protein, and used at 1:15,000 dilution.

Conformation-sensitive (non-denaturing) gels were done as described^27^,^46^. Precultures of test strains were grown to OD_600_=0.5 in BHI medium at 37 °C. They were then split in equal aliquots, and further grown for 4 h in BHI or BHI-FA-T. Cultures were harvested for preparation of protein extracts as described^27^ from the equivalent of OD_600_=5. DTT-treated extracts from BHI-grown *S. aureus* Newman were used as reference for the position of holo-ACP^27^,^47^. Samples (equivalent to OD_600_=0.25 in 10 μl) were loaded on 13% polyacrylamide gels containing 1 M urea, which were run at 100 volts for 3 h, and then transferred to PVDF membranes (0.2 μm; BioRad) for Western blotting and exposure using an ECL kit (Perkin-Elmer) as per supplier's instructions.

### Ethics statement

Animal experiments were performed at the animal facilities of HUPC in strict accordance with European Union guidelines for handling of laboratory animals, approved by the Ethics Committee for Animal Experimentation at the University of Paris-Descartes (CEEA 34), registration number 14-077.

### Data availability

Genome sequence data for DepT^R^-5, DepT^R^-72, DepT^R^-45, CondT^R^-17, and our parental Newman strain have been deposited in the European Nucleotide Archive and can be accessed at http://www.ebi.ac.uk/ena/data/view/PRJEB11476. The authors declare that all other data supporting the findings of this study are available within the article and its Supplementary Information files, or from the corresponding author upon request.

## Additional information

**How to cite this article:** Morvan, C. *et al*. Environmental fatty acids enable emergence of infectious *Staphylococcus aureus* resistant to FASII-targeted antimicrobials. *Nat. Commun.* **7,** 12944 doi: 10.1038/ncomms12944 (2016).

## Supplementary Material

Supplementary Information Supplementary Figures 1-7 and Supplementary References

## Figures and Tables

**Table 1:** Characterization of FA-T^R^ mutants.

**Strain**	Fatty acid prototroph/dependent*	**Fatty acid profile in FA-T**†	**Detected FASII mutation**	**Other mutations**‡
Newman^#^	Prototroph	–	–	
EndoT^R^-62	Prototroph	Endo	FabI^A95V^ Nt 977098 C→T	
EndoT^R^-41	Prototroph	Endo	FabI^I193N^ Nt 977392 T→A	
CondT^R^-1	Prototroph	Exo	FabD^G196V^ Nt 1250192 G→T	
CondT^R^-4	Prototroph	Exo	FabD^G196V^ Nt 1250192 G→T	
CondT^R^-7	Prototroph	Exo	FabD^G196V^ Nt 1250192 G→T	
CondT^R^-83	Prototroph	Exo	FabD^Q265P^ Nt 1250399 A→C	
CondT^R^-17^#^	Prototroph	Exo	FabD^G196R^ Nt 1250191 G→C	None detected
DepT^R^-5^#^	Dependent	Exo	FabD^Trunc AA8+14AA tail^ Nt 1249630 T insertion	None detected
DepT^R^-45^#^	Dependent	Exo	FabD^Trunc AA 285^ Nt 1250461 A deletion	NWMN_1735§ Nt 1935749 T deletion
DepT^R^-72^#^	Dependent	Exo	FabD^Trunc AA289+3AA tail^ Nt 1250471 A deletion	NWMN_0043§ Nt 56530 G deletion
DepT^R^-28	Dependent	Exo	AccB^D25Y^ Nt 1603874 G→T	
DepT^R^-71	Dependent	Exo	FabD^Trunc AA 147^ Nt 1250042 C→A	

^*^Prototrophs retain fatty acid synthesis capacity; in the absence of fatty acids they display endogenous (Endo) profiles; in the presence of fatty acids and a FASII inhibitor, they display either endogenous (Endo) or exogenous (Exo) profiles. Fatty-acid-dependent (DepT^R^) strains rely on exogenous fatty acids under all conditions.

^†^Fatty acid profiles were determined from cultures in BHI-FA-T (C14:0, C16:0 and C18:1, 0.17 mM each, and 0.25 μg ml^−1^ triclosan in BHI medium). # after strain names signify that strains were genome sequenced.

^‡^Other mutated genes were detected among the genome-sequenced isolates (strains designated with #).

^§^NWMN_1735 encodes a 978 AA ORF with partial similarity to DNA repair proteins^40^. A single nucleotide deletion (position 1935749) interrupts the ORF at position 428, adding a 5 AA out-of-frame tail. NWMN_0043 encodes Conserved Staphylococcal Antigen 1b, Csa1b, one of a conserved family of 18 protein homologues present in the Newman strain^39^. A single nucleotide deletion (position 56530) truncates the 256 AA ORF after position 82, with a 3 AA out-of-frame tail. All strains exhibiting exogenous fatty acid profiles encode wild type *fabI* loci. Nt, nucleotide; trunc, truncated.

**Table 2:** Natural clinical isolates bear *fabD* variants that favour FASII bypass.

**Strain**	**Origin**	**FabD196 locus**	**Growth on**		**FA profile on FA-T**
			**BHI**	**FA-T**	
Newman	Clinical-lab	FabD^G196^	++++	–	–
SA957	Blood	FabD^G196^	++++	–	–
SA40	Nare	FabD^S196^	++++	++++	Exo
MO251	Sputum	FabD^R196^	++++	+	Exo
MO1532	Nare	FabD^R196^	++++	++++	Exo
CondT^R^-4	FAT^R^	FabD^V196^	++++	+++	Exo
CondT^R^-17	FAT^R^	FabD^R196^	++++	+++	Exo

The NCBI database was searched for natural *S. aureus* isolates bearing FabD proteins with residues other than G196. Three such strains were received: CC5 isolate M0251, M1532 (Brigham and Women's Hospital, Boston; Mary Delaney and Michael Calderwood), and ST59 strain SA40 a Taiwanese commensal strain (Götz and Stemmler;^45^). ST59 strain SA957, whose *fabD* is identical to that of SA40 except for position 196, was used as reference for SA40 (Götz and Stemmler^45^). All strains listed above were grown overnight in BHI, BHI-FA (not presented) and BHI-FA-T. Overnight OD_600_: ++++=8 or above; +++=3–7.9; ++=1 to 2.9; +=0.5–1; −=no growth. Growth results were within this range in three biological replicates. Fatty acid profiles for all strains but Newman and SA957 were determined on BHI-FA-T. FA, fatty acid; FA-T, BHI-FA-T medium. Exo, exogenous fatty acid profile.

## References

[b1] Lowy F. D. Methicillin-resistant *Staphylococcus aureus*: where is it coming from and where is it going? JAMA Internal Med. 173, 1978–1979 (2013). 24042964 10.1001/jamainternmed.2013.8277

[b2] Uhlemann A. C., Otto M., Lowy F. D. & DeLeo F. R. Evolution of community- and healthcare-associated methicillin-resistant *Staphylococcus aureus*. Infect. Genet. Evol. 21, 563–574 (2014). 23648426 10.1016/j.meegid.2013.04.030 PMC3884050

[b3] Banevicius M. A., Kaplan N., Hafkin B. & Nicolau D. P. Pharmacokinetics, pharmacodynamics and efficacy of novel FabI inhibitor AFN-1252 against MSSA and MRSA in the murine thigh infection model. J. Chemother. 25, 26–31 (2013). 23433441 10.1179/1973947812Y.0000000061 PMC3558988

[b4] Escaich S. . The MUT056399 inhibitor of FabI is a new antistaphylococcal compound. Antimicrob. Agents Chemother. 55, 4692–4697 (2011). 21825292 10.1128/AAC.01248-10 PMC3186954

[b5] Feng Z., Chakraborty D., Dewell S. B., Reddy B. V. & Brady S. F. Environmental DNA-encoded antibiotics fasamycins A and B inhibit FabF in type II fatty acid biosynthesis. J. Am. Chem. Soc. 134, 2981–2987 (2012). 22224500 10.1021/ja207662w PMC3335777

[b6] Moir D. T., Opperman T. J., Butler M. M. & Bowlin T. L. New classes of antibiotics. Curr. Opin. Pharmacol. 12, 535–544 (2012). 22841284 10.1016/j.coph.2012.07.004

[b7] Park H. S. . Antistaphylococcal activities of CG400549, a new bacterial enoyl-acyl carrier protein reductase (FabI) inhibitor. J. Antimicrob. Chemother. 60, 568–574 (2007). 17606482 10.1093/jac/dkm236

[b8] Park H. S. . CG400462, a new bacterial enoyl-acyl carrier protein reductase (FabI) inhibitor. Int. J. Antimicrob. Agents 30, 446–451 (2007). 17723291 10.1016/j.ijantimicag.2007.07.006

[b9] Schiebel J. . Rational design of broad spectrum antibacterial activity based on a clinically relevant enoyl-acyl carrier protein (ACP) reductase inhibitor. J. Biol. Chem. 289, 15987–16005 (2014). 24739388 10.1074/jbc.M113.532804 PMC4047375

[b10] Wang J. . Discovery of platencin, a dual FabF and FabH inhibitor with *in vivo* antibiotic properties. Proc. Natl Acad. Sci. USA 104, 7612–7616 (2007). 17456595 10.1073/pnas.0700746104 PMC1863502

[b11] Wang J. . Platensimycin is a selective FabF inhibitor with potent antibiotic properties. Nature 441, 358–361 (2006). 16710421 10.1038/nature04784

[b12] McMurry L. M., Oethinger M. & Levy S. B. Triclosan targets lipid synthesis. Nature 394, 531–532 (1998). 9707111 10.1038/28970

[b13] Webster J., Faoagali J. L. & Cartwright D. Elimination of methicillin-resistant *Staphylococcus aureus* from a neonatal intensive care unit after hand washing with triclosan. J. Paediatr. Child Health 30, 59–64 (1994). 8148192 10.1111/j.1440-1754.1994.tb00568.x

[b14] Bamber A. I. & Neal T. J. An assessment of triclosan susceptibility in methicillin-resistant and methicillin-sensitive *Staphylococcus aureus*. J. Hosp. Infect. 41, 107–109 (1999). 10063472 10.1016/s0195-6701(99)90047-6

[b15] Bhargava H. N. & Leonard P. A. Triclosan: applications and safety. Am. J. Infect. Control 24, 209–218 (1996). 8807001 10.1016/s0196-6553(96)90017-6

[b16] Brenwald N. P. & Fraise A. P. Triclosan resistance in methicillin-resistant *Staphylococcus aureus* (MRSA). J. Hosp. Infect. 55, 141–144 (2003). 14529640 10.1016/s0195-6701(03)00222-6

[b17] Cherednichenko G. . Triclosan impairs excitation-contraction coupling and Ca^2+^ dynamics in striated muscle. Proc. Natl Acad. Sci. USA 109, 14158–14163 (2012). 22891308 10.1073/pnas.1211314109 PMC3435154

[b18] Syed A. K., Ghosh S., Love N. G. & Boles B. R. Triclosan promotes *Staphylococcus aureus* nasal colonization. MBio 5, e01015 (2014). 10.1128/mBio.01015-13 PMC3993860 24713325

[b19] Yueh M. F. . The commonly used antimicrobial additive triclosan is a liver tumor promoter. *Proc. Natl Acad. Sci. USA* (2014). 10.1073/pnas.1419119111 PMC4260592 25404284

[b20] Ciusa M. L. . A novel resistance mechanism to triclosan that suggests horizontal gene transfer and demonstrates a potential selective pressure for reduced biocide susceptibility in clinical strains of *Staphylococcus aureus*. Int. J. Antimicrob. Agents 40, 210–220 (2012). 22789727 10.1016/j.ijantimicag.2012.04.021

[b21] Balemans W. . Essentiality of FASII pathway for *Staphylococcus aureus*. Nature 463, E3–E4 (2010). 20090698 10.1038/nature08667

[b22] Kaplan N., Garner C. & Hafkin B. AFN-1252 *in vitro* absorption studies and pharmacokinetics following microdosing in healthy subjects. Eur. J. Pharm. Sci. 50, 440–446 (2013). 23988847 10.1016/j.ejps.2013.08.019

[b23] Parsons J. B. & Rock C. O. Is bacterial fatty acid synthesis a valid target for antibacterial drug discovery? Curr. Opin. Microbiol. 14, 544–549 (2011). 21862391 10.1016/j.mib.2011.07.029 PMC3193581

[b24] Schiebel J. . *Staphylococcus aureus* FabI: inhibition, substrate recognition, and potential implications for *in vivo* essentiality. Structure 20, 802–813 (2012). 22579249 10.1016/j.str.2012.03.013 PMC3376755

[b25] Brinster S. . Type II fatty acid synthesis is not a suitable antibiotic target for Gram-positive pathogens. Nature 458, 83–86 (2009). 19262672 10.1038/nature07772

[b26] Brinster S. . Essentiality of FASII pathway for *Staphylococcus aureus*. Nature 463, E4–E5 (2010). 10.1038/nature08667 20090698

[b27] Parsons J. B., Frank M. W., Subramanian C., Saenkham P. & Rock C. O. Metabolic basis for the differential susceptibility of Gram-positive pathogens to fatty acid synthesis inhibitors. Proc. Natl. Acad. Sci. USA 108, 15378–15383 (2011). 21876172 10.1073/pnas.1109208108 PMC3174620

[b28] Zhu L. . The two functional enoyl-acyl carrier protein reductases of Enterococcus faecalis do not mediate triclosan resistance. MBio 4, e00613–e00613 (2013). 10.1128/mBio.00613-13 PMC3791895 24085780

[b29] Kaplan N. . Mode of action, *in vitro* activity, and *in vivo* efficacy of AFN-1252, a selective antistaphylococcal FabI inhibitor. Antimicrob. Agents Chemother. 56, 5865–5874 (2012). 22948878 10.1128/AAC.01411-12 PMC3486558

[b30] Parsons J. B. . Perturbation of *Staphylococcus aureus* gene expression by the enoyl-acyl carrier protein reductase inhibitor AFN-1252. Antimicrob. Agents Chemother. 57, 2182–2190 (2013). 23459481 10.1128/AAC.02307-12 PMC3632907

[b31] Parsons J. B. . Identification of a two-component fatty acid kinase responsible for host fatty acid incorporation by *Staphylococcus aureus*. Proc. Natl Acad. Sci. USA 111, 10532–10537 (2014). 25002480 10.1073/pnas.1408797111 PMC4115530

[b32] Parsons J. B., Frank M. W., Rosch J. W. & Rock C. O. *Staphylococcus aureus* fatty acid auxotrophs do not proliferate in mice. Antimicrob. Agents Chemother. 57, 5729–5732 (2013). 23979734 10.1128/AAC.01038-13 PMC3811263

[b33] Coleman J. Characterization of the *Escherichia coli* gene for 1-acyl-sn-glycerol-3-phosphate acyltransferase (plsC). Mol. Gen. Genet. 232, 295–303 (1992). 1557036 10.1007/BF00280009

[b34] Parsons J. B., Yao J., Jackson P., Frank M. & Rock C. O. Phosphatidylglycerol homeostasis in glycerol-phosphate auxotrophs of *Staphylococcus aureus*. BMC Microbiol. 13, 260 (2013). 24238430 10.1186/1471-2180-13-260 PMC3840577

[b35] Nakamura T. . Serum fatty acid levels, dietary style and coronary heart disease in three neighbouring areas in Japan: the Kumihama study. Br. J. Nutr. 89, 267–272 (2003). 12575911 10.1079/BJN2002747

[b36] Ni Raghallaigh S., Bender K., Lacey N., Brennan L. & Powell F. C. The fatty acid profile of the skin surface lipid layer in papulopustular rosacea. Br. J. Dermatol. 166, 279–287 (2012). 21967555 10.1111/j.1365-2133.2011.10662.x

[b37] Greenway D. L. & Dyke K. G. Mechanism of the inhibitory action of linoleic acid on the growth of *Staphylococcus aureus*. J. Gen. Microbiol. 115, 233–245 (1979). 93615 10.1099/00221287-115-1-233

[b38] Parsons J. B., Yao J., Frank M. W., Jackson P. & Rock C. O. Membrane disruption by antimicrobial fatty acids releases low-molecular-weight proteins from *Staphylococcus aureus*. J. Bacteriol. 194, 5294–5304 (2012). 22843840 10.1128/JB.00743-12 PMC3457211

[b39] Schluepen C. . Mining the bacterial unknown proteome: identification and characterization of a novel family of highly conserved protective antigens in *Staphylococcus aureus*. Biochem. J. 455, 273–284 (2013). 23895222 10.1042/BJ20130540

[b40] Krishnamurthy M., Tadesse S., Rothmaier K. & Graumann P. L. A novel SMC-like protein, SbcE (YhaN), is involved in DNA double-strand break repair and competence in *Bacillus subtilis*. Nucleic Acids Res. 38, 455–466 (2010). 19906728 10.1093/nar/gkp909 PMC2811018

[b41] Hong S. K. . New design platform for malonyl-CoA-acyl carrier protein transacylase. FEBS Lett. 584, 1240–1244 (2010). 20176020 10.1016/j.febslet.2010.02.038

[b42] Serre L., Verbree E. C., Dauter Z., Stuitje A. R. & Derewenda Z. S. The *Escherichia coli* malonyl-CoA:acyl carrier protein transacylase at 1.5-A resolution. Crystal structure of a fatty acid synthase component. J. Biol. Chem. 270, 12961–12964 (1995). 7768883 10.1074/jbc.270.22.12961

[b43] Parsons J. B., Frank M. W., Jackson P., Subramanian C. & Rock C. O. Incorporation of extracellular fatty acids by a fatty acid kinase-dependent pathway in *Staphylococcus aureus*. Mol. Microbiol. 92, 234–245 (2014). 24673884 10.1111/mmi.12556 PMC4007170

[b44] Hafkin B., Kaplan N. & Murphy B. Efficacy and safety of AFN-1252, the first staphylococcus-specific antibacterial agent, in the treatment of ABSSSI, including patients with significant co-morbidities. Antimicrob. Agents Chemother. 60, 1695–1701 (2015). 26711777 10.1128/AAC.01741-15 PMC4775962

[b45] Chen C. J. . Characterization and comparison of 2 distinct epidemic community-associated methicillin-resistant *Staphylococcus aureus* clones of ST59 lineage. PLoS One 8, e63210 (2013). 24039691 10.1371/journal.pone.0063210 PMC3764004

[b46] Cronan J. E. & Thomas J. Bacterial fatty acid synthesis and its relationships with polyketide synthetic pathways. Methods Enzymol. 459, 395–433 (2009). 19362649 10.1016/S0076-6879(09)04617-5 PMC4095770

[b47] Masoudi A., Raetz C. R., Zhou P. & Pemble C. W. T. Chasing acyl carrier protein through a catalytic cycle of lipid A production. Nature 505, 422–426 (2014). 24196711 10.1038/nature12679 PMC3947097

[b48] Kaplan N. . *In vitro* activity (MICs and rate of kill) of AFN-1252, a novel FabI inhibitor, in the presence of serum and in combination with other antibiotics. J. Chemother. 25, 18–25 (2013). 23433440 10.1179/1973947812Y.0000000063 PMC3558989

[b49] Yum J. H. . *In vitro* activities of CG400549, a novel FabI inhibitor, against recently isolated clinical staphylococcal strains in Korea. Antimicrob. Agents Chemother. 51, 2591–2593 (2007). 17420210 10.1128/AAC.01562-06 PMC1913239

[b50] Ruch F. E. & Vagelos P. R. The isolation and general properties of *Escherichia coli* malonyl coenzyme A-acyl carrier protein transacylase. J. Biol. Chem. 248, 8086–8094 (1973). 4584822

[b51] Albanesi D. . Structural basis for feed-forward transcriptional regulation of membrane lipid homeostasis in *Staphylococcus aureus*. PLoS Pathog. 9, e1003108 (2013). 23300457 10.1371/journal.ppat.1003108 PMC3536700

[b52] Schujman G. E., Paoletti L., Grossman A. D. & de Mendoza D. FapR, a bacterial transcription factor involved in global regulation of membrane lipid biosynthesis. Dev. Cell 4, 663–672 (2003). 12737802 10.1016/s1534-5807(03)00123-0

[b53] Martinez M. A. . A novel role of malonyl-ACP in lipid homeostasis. Biochemistry 49, 3161–3167 (2010). 20201588 10.1021/bi100136n

[b54] Do T. Q. . Lipids including cholesteryl linoleate and cholesteryl arachidonate contribute to the inherent antibacterial activity of human nasal fluid. J. Immunol. 181, 4177–4187 (2008). 18768875 10.4049/jimmunol.181.6.4177 PMC2597438

[b55] Lin Y. T. . Emergence of a small colony variant of vancomycin-intermediate *Staphylococcus aureus* in a patient with septic arthritis during long-term treatment with daptomycin. J. Antimicrob. Chemother. 71, 1807–1814 (2016). 26968883 10.1093/jac/dkw060

[b56] Kubota N. . First isolation of oleate-dependent *Enterococcus faecalis* small-colony variants from the umbilical exudate of a paediatric patient with omphalitis. J. Med. Microbiol. 62, 1883–1890 (2013). 24072765 10.1099/jmm.0.062752-0

[b57] Nicolaides N. Skin lipids: their biochemical uniqueness. Science 186, 19–26 (1974). 4607408 10.1126/science.186.4158.19

[b58] Becker L. C. . Final report of the amended safety assessment of myristic acid and its salts and esters as used in cosmetics. Int. J. Toxicol. 29, 162S–186S (2010). 20634506 10.1177/1091581810374127

[b59] Fitzgerald J. R. Evolution of *Staphylococcus aureus* during human colonization and infection. Infect. Genet. Evol. 21, 542–547 (2014). 23624187 10.1016/j.meegid.2013.04.020

[b60] Herbert S. . Repair of global regulators in *Staphylococcus aureus* 8325 and comparative analysis with other clinical isolates. Infect. Immun. 78, 2877–2889 (2010). 20212089 10.1128/IAI.00088-10 PMC2876537

[b61] Blattner F. R. . The complete genome sequence of *Escherichia coli* K-12. Science 277, 1453–1462 (1997). 9278503 10.1126/science.277.5331.1453

[b62] Yamamoto Y. . The Group B Streptococcus NADH oxidase Nox-2 is involved in fatty acid biosynthesis during aerobic growth and contributes to virulence. Mol. Microbiol. 62, 772–785 (2006). 16999835 10.1111/j.1365-2958.2006.05406.x

[b63] Gibson D. G. . Enzymatic assembly of DNA molecules up to several hundred kilobases. Nat. Methods 6, 343–345 (2009). 19363495 10.1038/nmeth.1318

[b64] Lartigue M. F. & Bouloc P. A tetracycline-inducible expression vector for *Streptococcus agalactiae* allowing controllable gene expression. J. Microbiol. Methods 96, 16–18 (2014). 24200710 10.1016/j.mimet.2013.10.020

[b65] Kraemer G. R. & Iandolo J. J. High-frequency transformation of *Staphylococcus aureus* by electroporation. Curr. Microbiol. 21, 373–376 (1990).

[b66] Post-Beittenmiller D., Jaworski J. G. & Ohlrogge J. B. *In vivo* pools of free and acylated acyl carrier proteins in spinach. Evidence for sites of regulation of fatty acid biosynthesis. J. Biol. Chem. 266, 1858–1865 (1991). 1988450

[b67] Zhang Y. M. & Rock C. O. Membrane lipid homeostasis in bacteria. Nat. Rev. Microbiol. 6, 222–233 (2008). 18264115 10.1038/nrmicro1839

