# Supplementary material for: Environmental fatty acids enable emergence of infectious Staphylococcus aureus resistant to FASII-targeted antimicrobials
Source: Nat Commun. 2016 Oct 5;7:12944. doi: 10.1038/ncomms12944 (PMC5059476; doi:10.1038/ncomms12944)
Supplement: Supplementary Information — Supplementary Figures 1-7 and Supplementary References [file ncomms12944-s1.pdf]

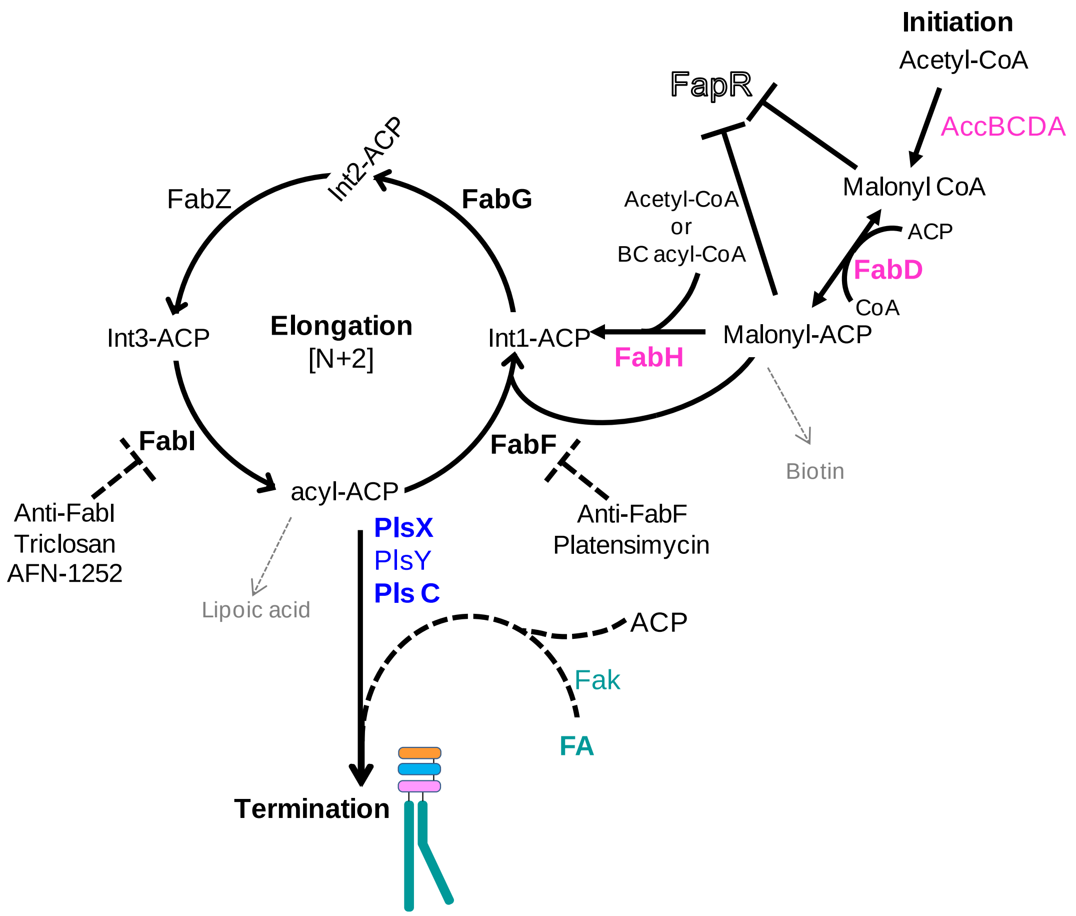

Supplementary Fig. 1. FASII pathway in *S. aureus* and anti-FASII drug targets

FASII comprises an initiation step (enzymes in magenta), in which Acc carboxylase (AccBCDA) uses acetyl-CoA to produce malonyl-CoA, FabD transacylase transfers the malonyl group from CoA to ACP (acyl carrier protein), and FabH (β-ketoacyl-ACP synthase III) joins either acetyl-CoA and malonyl-ACP to form acetoacetyl-ACP, or alternatively uses branched chain (BC) acyl-CoA (formed from branched chain amino acids*)* instead of acetyl-CoA to condense with malonyl-ACP: this leads to branched chain FA formation. The acyl-ACP substrates then enter a cycle of recursive FA synthesis. FapR is a repressor of both FASII and phospholipid synthesis genes (*fabD*, *fabH*, *fabG*, *fabI*, *fabF*, *plsX*, and *plsC* targets are in bold) [1]. FapR repression is alleviated by malonyl-CoA and malonyl-ACP [1-3].

The FA elongation cycle (enzymes in black) comprises four consecutively acting enzymes; Int1-ACP to Int3-ACP represent reaction intermediates. FabG, the β-ketoacyl-ACP reductase, uses either FabH initiation products, or already elongated FA intermediates (FabF products) as substrates. FabZ dehydrates the FA intermediate, and FabI enoyl reductase completes the cycle to form acyl-ACP, which either continues in the cycle to add a new 2-carbon malonyl group (from malonyl-ACP) *via* FabF, or is used for phospholipid synthesis by Pls enzymes (blue). The described steps are characterized in previous work [4,5]. FASII may accept non-conventional substrates, i.e., for biotin or lipoic acid synthesis (grey) [6,7]. FabI inhibitors triclosan, AFN-1252, and FabF inhibitor platensimycin are used in this work.

Fak genes (turquoise) are essential for phosphorylation of exogenous fatty acids, the first step in their incorporation in phospholipids [8]. PlsY can then join the phosphorylated fatty acid to position 1 of the glycerol-phosphate backbone. An alternative possibility is that the phosphorylated fatty acid is used by PlsX in a reverse reaction that converts it to long-chain acyl-ACP, which would then be joined to position 2 of the glycerol-phosphate backbone by PlsC [8]. The present work provides evidence for the latter reaction in *S. aureus* (see Fig. 7).

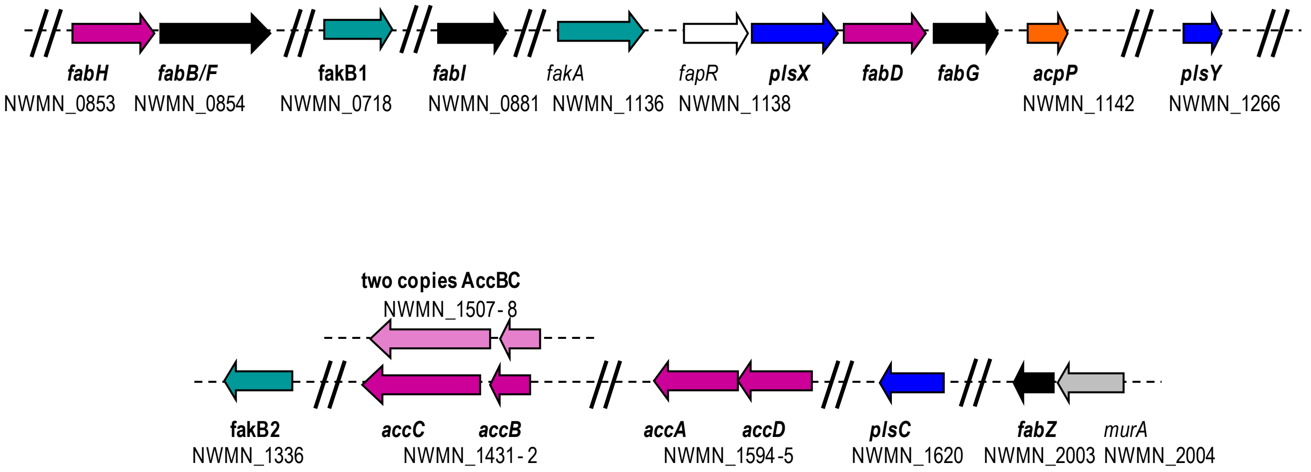

Supplementary Fig. 2. Organization of known FASII and associated genes in *S. aureus* Newman

Known FASII and associated genes leading to phospholipid synthesis are depicted. Magenta, genes involved in FASII initiation; Black, the recursive elongation cycle; Blue, termination and synthesis of phosphatidic acid (see [5] for review); turquoise, *fak* genes identified as needed for exogenous FA incorporation; *fakA*, *fakB1* (C14 and C16 –specific) and *fakB2* (C18:1 –specific) phosphorylate exogenous FA in *S. aureus*, mediating their utilization for phospholipid synthesis [8]. White, FapR regulator of FASII and phospholipid synthesis genes [1]; grey, non-FASII gene in an operon with *fabZ.* Two copies of AccBC appear to be present on the Newman genome; the role of NWMN_1507/NWMN_1508 is unknown. Orange, *acpP*, encodes ACP which is required in major steps throughout FASII and phospholipid synthesis.

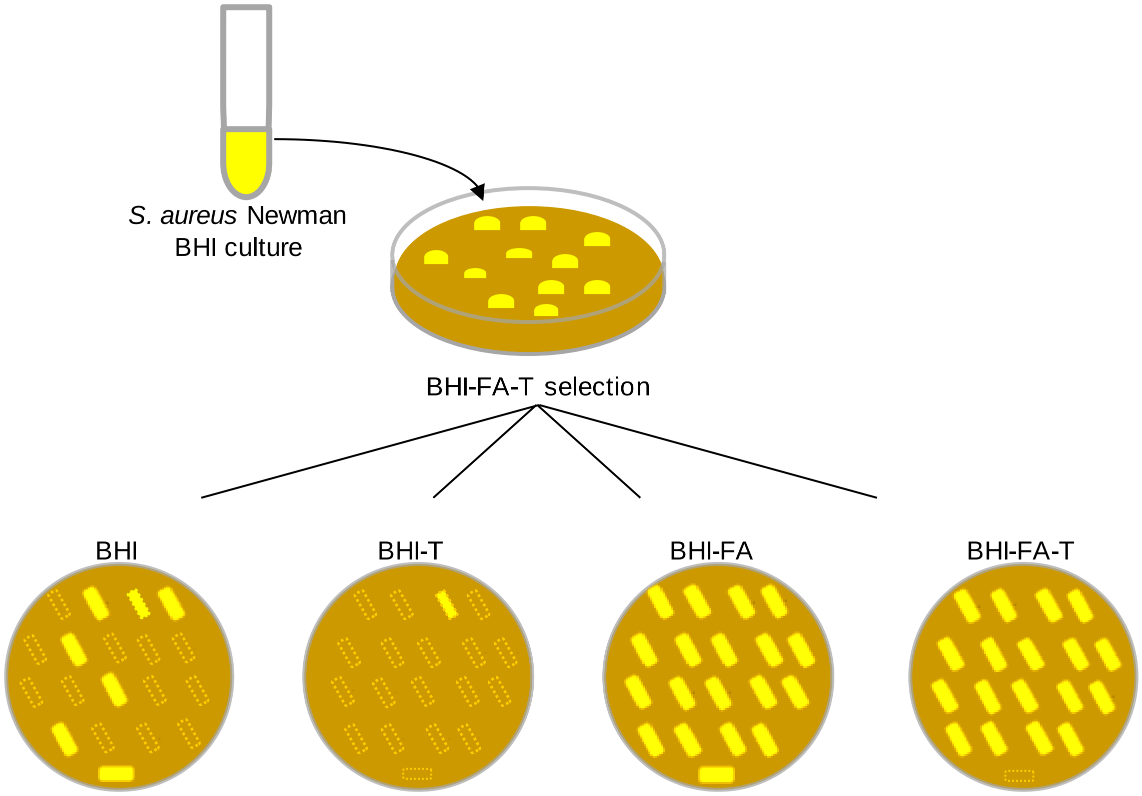

	BHI	BHI-FA	BHI-T	BHI-FA-T
Triclosan-resistant	+	+	+	+
FA-dependent for growth and triclosan-resistance (DepT^R)	-	+	-	+
Normal growth, uses FA for triclosan-resistance (CondT^R)	+	+	-	+

Supplementary Fig. 3. Experimental design for identification of FASII bypass mutants
Dilutions of overnight BHI cultures of *S. aureus* Newman strain are plated on BHI-FA-T medium and incubated at 37°C for 48 hours. FA-T^R-resistant colonies are repicked to BHI, BHI-FA, BHI-T, and BHI-FA-T medium, containing as appropriate fatty acids C14:0, C16:0 and C18:1 (0.17 mM each), and triclosan (0.25 µg/mL). The horizontal streak at the bottom of plates corresponds to the parental Newman strain. Plate interpretation is indicated in Table below. Phenotypes of candidate mutants are confirmed after clone purifications. Note that proportions of FA-dependent and conditional mutants varied between experiments and duration of incubation of selection plates. In the absence of fatty acids, DepT^R clones grow poorly or not at all , whereas CondT^R clones grow normally.

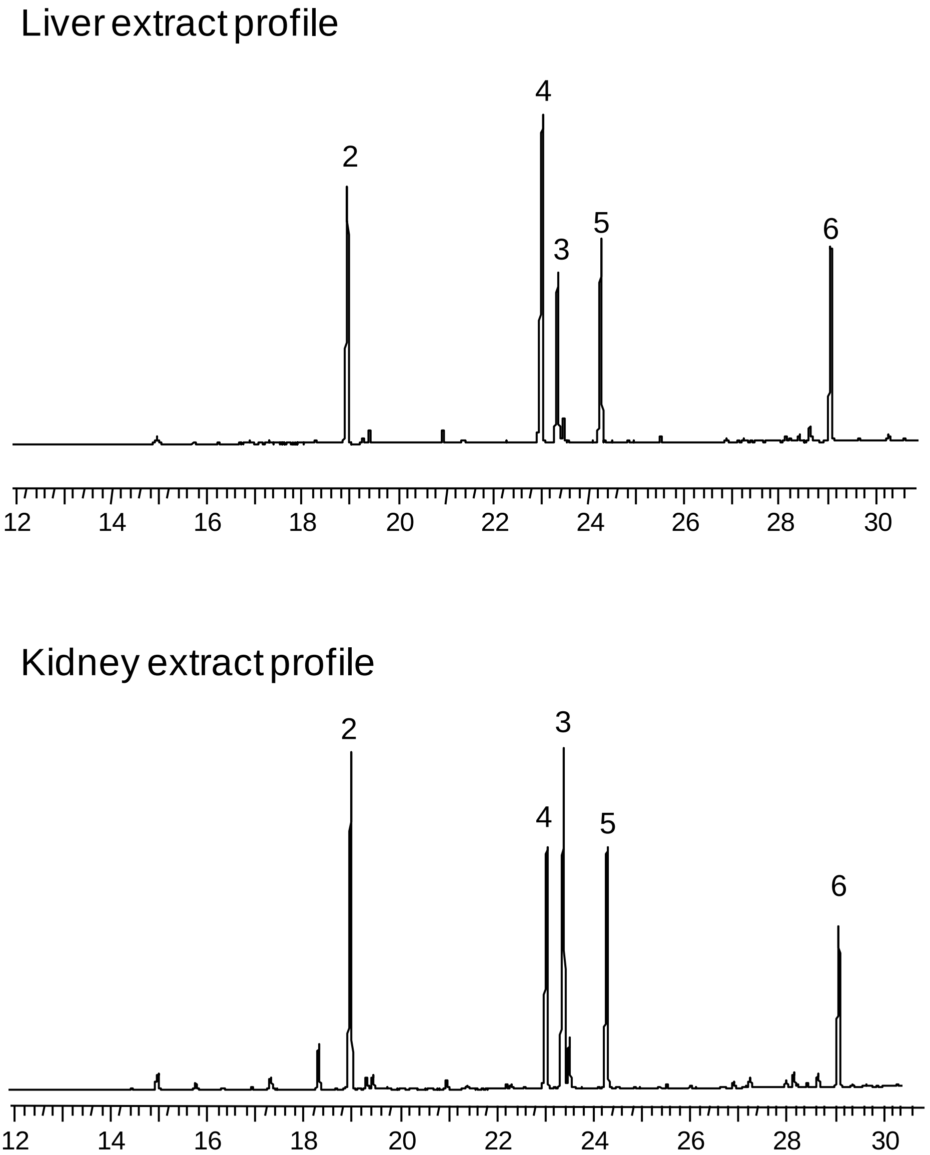

Supplementary Fig. 4. Fatty acid profiles of liver and kidney extracts used as fatty acid supplements

Organ preparation and extracts for gas chromatography are described in Methods. Fatty acids are: 2- C16:0; 3- C18:1; 4- C18:0; 5- C18:2; 6- C20:4.

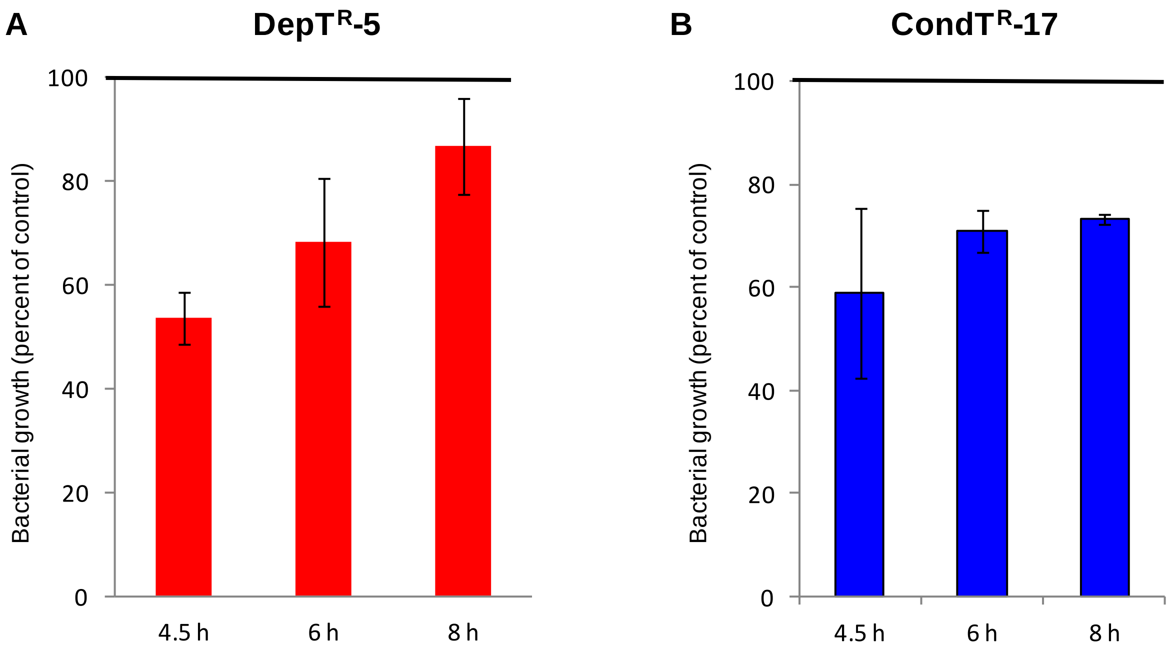

Supplementary Fig. 5. Complementation of DepT^R-5 and CondT^R-17 *fabD* mutants by wild type *fabD* leads to a growth lag in triclosan

Experimental conditions are as in Fig. 4. Histograms above represent the relative growth at different time points, of FA-T^R (pJ-fabD) mutants compared to the respective control strain FA-T^R (pJ), grown in LB-triclosan with kidney extract. Ratios are determined at the indicated growth times for each of three independent experiments. **A**, Ratio of OD_{600} DepT^R-5 (pJ-FabD) to Dep-5 (pJ); **B,** Ratio of OD_{600} CondT^R-17 (pJ-FabD) relative to Cond-17 (pJ). Growth of reference strains DepT^R-5 (pJ) and CondT^R-17 (pJ), is set at 100% (black line). The presence of pJ-FabD-carrying strains systematically showed a growth lag when triclosan was present, compared to the cognate strains carrying the control plasmid.

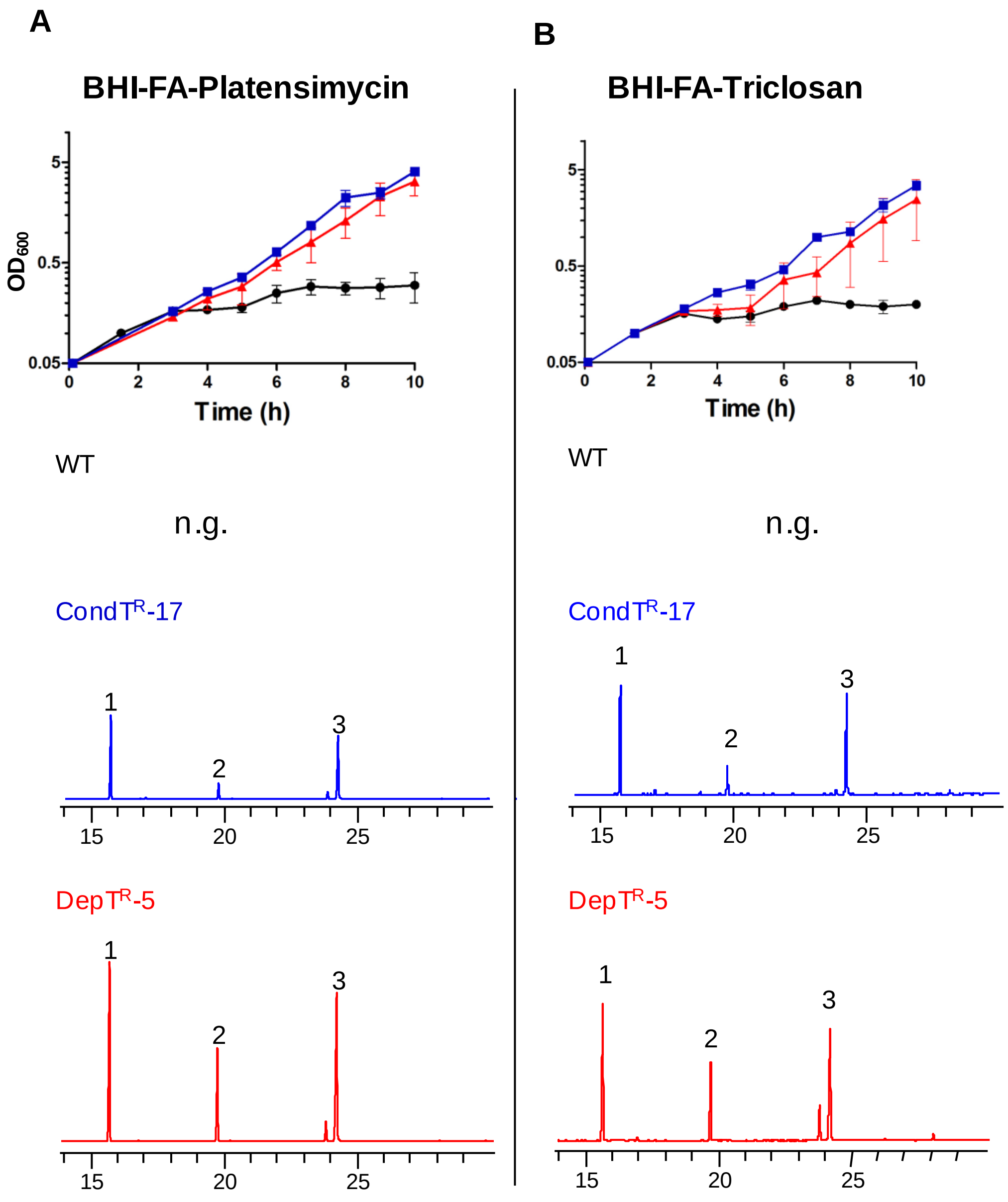

Supplementary Fig. 6. ***S. aureus*** **FA-T^{R} strains DepT^{R}-5 and CondT^{R}-17 bypass inhibitors of FabI and also FabF**
Newman WT, CondT^{R}-17, and DepT^{R}-5 strains were grown in BHI-FA containing **A**, platensimycin 2 µg/mL, or **B**, triclosan (0.25 µg/ml) for comparison. Results of WT and CondT^{R}-17 with triclosan in **B** are shown in Fig. 2 as experiments were performed at the same time. Growth curves (upper) show the average of two biologically independent duplicates. The range of OD_{600} for each time point is presented. Corresponding fatty acid profiles from the 10 H point (lower) of one experiment are shown. N.g., no growth. Endogenous branched-chain fatty acid (*ai*15:0) is indicated with arrows. Fatty acids are 1- C14:0; 2- C16:0; and 3- C18:1, the fatty acids comprised in BHI-FA-T medium. Black, WT; blue, CondT^{R}-17; red, DepT^{R}-5.

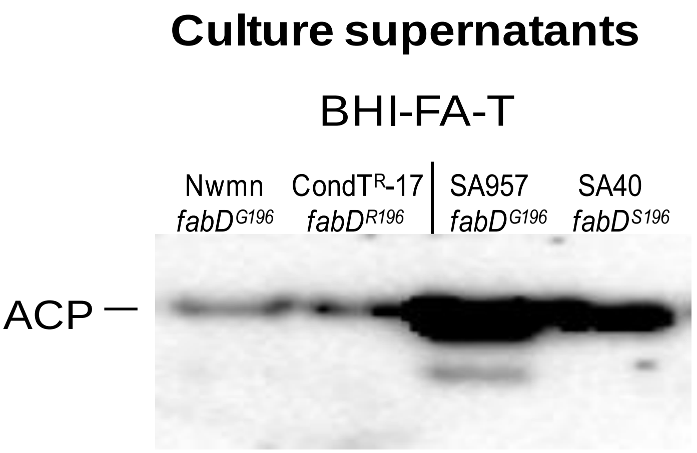

Supplementary Fig. 7. ACP release into supernatant is unaffected by *fabD*^{196} polymorphisms

Experiments were performed as described in Fig. 7 and in Methods. In the same experiments, supernatants were recovered, filter-sterilized, and concentrated ten-fold using Amicon Ultra-2ml membranes (3 Kd cutoff; Merck**,** France). Samples were treated with DTT (200 mM) to dissociate ACP from acyl groups. Amounts loaded on conformation-sensitive gels correspond to 0.25units of OD_{600} as for cell pellets (see **Fig. 7**). Anti-ACP antibodies were used to identify ACP moieties after gel transfer. The portion of the gel giving a signal with anti-ACP antibodies is shown. Nwmn is the parental strain for CondT^{R}-17; SA957 and SA40 are closely related clinical isolates belonging to ST59 [9]. For both pairs, the encoded FabD enzymes differ from their respective reference strains by a single change at amino acid position 196. The migration position of free ACP is indicated.

Supplementary References

1. Albanesi D, Reh G, Guerin ME, Schaeffer F, Debarbouille M, et al. Structural basis for feed-forward transcriptional regulation of membrane lipid homeostasis in *Staphylococcus aureus*. PLoS Pathog 9: e1003108 (2013).
2. Schujman, G.E., Paoletti, L., Grossman, A.D. & de Mendoza, D. FapR, a bacterial transcription factor involved in global regulation of membrane lipid biosynthesis. *Dev Cell* **4**, 663-72 (2003).
3. Martinez, M.A. *et al.* A novel role of malonyl-ACP in lipid homeostasis. *Biochemistry* **49**, 3161-7 (2010).
4. Schiebel J, Chang A, Lu H, Baxter MV, Tonge PJ, et al. *Staphylococcus aureus* FabI: inhibition, substrate recognition, and potential implications for in vivo essentiality. Structure 20: 802-813 (2012).
5. Zhang YM, Rock CO. Membrane lipid homeostasis in bacteria. Nat Rev Microbiol 6: 222-233 (2008).
6. Agarwal V, Lin S, Lukk T, Nair SK, Cronan JE. Structure of the enzyme-acyl carrier protein (ACP) substrate gatekeeper complex required for biotin synthesis. Proc Natl Acad Sci U S A 109: 17406-17411 (2012).
7. Cronan JE. Biotin and Lipoic Acid: Synthesis, Attachment and Regulation. Ecosal Plus 2014. (2014)
8. Parsons JB, Broussard TC, Bose JL, Rosch JW, Jackson P, et al. Identification of a two-component fatty acid kinase responsible for host fatty acid incorporation by *Staphylococcus aureus*. Proc Natl Acad Sci U S A 111: 10532-10537 (2014).
9. Chen, C.J. *et al.* Characterization and comparison of 2 distinct epidemic community-associated methicillin-resistant *Staphylococcus aureus* clones of ST59 lineage. *PLoS One* **8**, e63210 (2013).
